# DAMF-Net: A Dynamic Receptive Field Enhancement and Semantic-Guided Adaptive Modulated Fusion Network for Steel Surface Defect Segmentation

**DOI:** 10.3390/s26144360

**Published:** 2026-07-09

**Authors:** Dengbiao Liu, Zhennan Chen, Chong Zhang, Wenxi Cui, Kai Sun, Boxing Yue, Linhai Zhang, Cuiyun Li

**Affiliations:** College of Intelligent Equipment, Shandong University of Science and Technology, Tai’an 271019, China; skd993790@sdust.edu.cn (D.L.); 202483230063@sdust.edu.cn (Z.C.); 202483230080@sdust.edu.cn (C.Z.); 202383230006@sdust.edu.cn (W.C.); 202383230004@sdust.edu.cn (K.S.); 202583230069@sdust.edu.cn (B.Y.); 202583230066@sdust.edu.cn (L.Z.)

**Keywords:** surface defect segmentation, feature representation failure, scale adaptivity, adaptive modulated fusion

## Abstract

In the industrial strip steel production process, complex background interference and varying defect scales make existing semantic segmentation models prone to scale confusion and noise propagation issues during the feature representation stage, thereby limiting segmentation accuracy and stability. To address these problems, this paper proposes a steel surface defect segmentation network based on dynamic receptive field modulation and semantic-guided adaptive modulated fusion, termed DAMF-Net. First, in the encoding stage, a kernel selection fusion attention module (KSFA) is designed, which constructs multi-scale receptive field responses through cascaded depthwise convolution, and combines spatial average prior with channel-level competitive weights to perform adaptive gating modulation on bottleneck features, thereby enhancing the model’s ability to represent different scale defect structures; second, in the decoding stage, a task-oriented Adaptive Modulated Fusion Module (AMFM), inspired by modulation-based feature fusion, is introduced to adaptively fuse shallow detail features and deep semantic features through branch-wise competitive weighting for each channel, thereby reducing the propagation of shallow background noise during decoding; additionally, a hybrid optimization objective combining binary cross-entropy and Dice loss is constructed to enhance the model’s learning ability for small-scale and low-contrast defects. Experimental results on the Severstal and ESDIs-SOD datasets show that the proposed method improves the mDice/Dice metrics by 2.38% and 4.40% respectively compared to the U-Net baseline, and exhibits lower prediction errors and more stable segmentation performance under complex background conditions. Meanwhile, DAMF-Net achieves an inference speed of 55.8 FPS while ensuring improved accuracy, demonstrating a good balance between precision and efficiency. This method provides an effective solution for high-precision segmentation of steel surface defects in complex industrial scenarios.

## 1. Introduction

Steel materials are widely used in key areas such as construction, bridges, automotive manufacturing, rail transit, and high-end equipment. The surface quality of steel not only affects the appearance of products but also directly relates to the material’s corrosion resistance, fatigue strength, and service safety [1]. During continuous rolling, heat treatment, and transportation processes, factors such as raw material fluctuations, equipment wear, and process disturbances can easily cause various defects on the steel surface, including scratches, cracks, inclusions, scale, and spalling [2]. These defects reduce product grade and economic value, and in severe cases, they may even lead to subsequent failures. Therefore, automatic detection and precise segmentation of steel surface defects have always been important research topics in industrial visual inspection [3]. Recent surveys on real-world industrial defect detection further indicate that modern manufacturing systems increasingly require inspection methods with high accuracy, automation, scalability, and adaptability to practical production environments [4]. In addition, steel surface defect analysis studies have emphasized that surface defects may cause not only aesthetic degradation but also deterioration of material performance, further highlighting the necessity of automated inspection [2].

Compared to natural scene images, the segmentation of surface defects on steel has more prominent industrial complexity. First, defects vary significantly in scale, morphology, and distribution, ranging from extremely fine linear scratches to large-scale irregular exfoliation areas, making it difficult for single-scale feature extraction to simultaneously capture both local details and overall structures [5]. Recent steel defect segmentation studies have also shown that edge-aware modeling, deformable feature fusion, and multi-scale feature fusion are effective for handling irregular defect shapes, improving boundary localization, and enhancing defect representation under complex surface conditions [6,7]. Secondly, in industrial environments, issues such as water stains, oil residues, scaling, and uneven brightness can closely resemble actual defects visually. This can easily lead to both false positives and missed detections [4]. Particularly in low-contrast and camouflaged defect scenarios, complex backgrounds further weaken the model’s stable segmentation ability for target areas [8]. Therefore, steel surface defect segmentation not only requires models to possess strong multi-scale representation capabilities but also demands good robustness and engineering practicality under complex backgrounds.

In recent years, deep learning has significantly advanced the technology for surface defect analysis. Ronneberger et al. [9] proposed U-Net, which achieved effective fusion of shallow spatial details and deep semantic information through an encoder–decoder structure and skip connections, becoming an important foundational framework for pixel-level segmentation tasks. Lin et al. [10] proposed FPN, which constructed a multi-scale feature pyramid through top-down path and lateral connections under self-top-down modeling, providing a general approach for identifying objects with significant scale variations. In industrial surface defect segmentation scenarios, Zhang et al. [11] proposed CAWANet, which enhanced feature expression in real-time segmentation through a context-aware weighted mechanism; Wang et al. [12] proposed Fast-SDNet, which achieved faster inference speeds with a more lightweight structure design under lower parameter conditions; Xiao et al. [13] proposed HMNet, which introduced state space modeling ideas into high-resolution surface defect segmentation to enhance long-range dependency representation capabilities; Sun et al. [14] proposed DEPANet, which enhanced defect boundary preservation and multi-scale fusion effects through edge-guided pyramid aggregation mechanisms; Ameri and Hsu [15] proposed LACTNet, which enhances local texture and global context representation capabilities through collaborative modeling of CNN and Transformer. These studies indicate that industrial defect segmentation is gradually evolving from direct Transformer of general segmentation frameworks to specialized optimization directions targeting complex backgrounds, multi-scale defects, and implementation deployment constraints. In addition, lightweight metal defect segmentation methods based on multi-scale feature fusion and knowledge distillation have recently been explored to balance segmentation accuracy and real-time prediction, indicating that efficient multi-scale representation remains an important research direction for industrial defect segmentation [7].

Beyond the split framework itself, attention modeling and adaptive fusion mechanisms also provide important support for industrial defect visual tasks. Hu et al. [16] proposed the SE module, which enhances effective feature response by explicitly modeling inter-channel dependencies. Woo et al. [17] proposed CBAM, combining channel attention with spatial attention to enable the network to focus on both key features and important regions simultaneously. Li et al. [18] proposed a selective kernel network, achieving dynamic receptive field adjustment through selective convolution kernels, providing an effective approach for multi-scale target representation. Dai et al. [19] proposed attention feature fusion, pointing out that cross-layer feature fusion should not rely solely on simple concatenation or element-wise addition, but should utilize adaptive weights to select and reconstruct information from different levels. Meanwhile, Ashrafi et al. [20] demonstrated through a systematic comparison of steel surface defect detection and segmentation methods that complex backgrounds, fine-grained structures, and implementation deployment constraints remain key directions for continuous optimization of current methods. Recent dynamic attention and hierarchical feature fusion methods for steel surface defect analysis further suggest that adaptive response selection is beneficial for improving the recognition of tiny defects and defects with large morphological variations [21,22].

Although existing methods have achieved positive progress, steel surface defect segmentation in complex industrial scenarios still faces two coupled challenges. First, defects usually exhibit significant scale and morphology variations, including tiny scratches, slender cracks, irregular patches, and large-area defects. Models relying on fixed receptive fields or static multi-scale structures may therefore suffer from scale ambiguity and incomplete feature representation. Second, shallow high-resolution features in encoder–decoder networks often contain background artifacts such as specular reflections, oxide-scale textures, water stains, and illumination noise. If these features are directly fused into the decoder through simple concatenation or addition, shallow noise may be propagated and amplified, leading to false positives and degraded boundary quality. This problem is consistent with recent observations in steel surface defect feature-fusion networks, where straightforward fusion of high-level semantic information and low-level detailed features may fail to fully exploit discriminative positional and semantic information [22]. To address these issues, this paper proposes DAMF-Net, an encoder–decoder network based on dynamic feature modulation and branch-wise competitive selection. The main idea is to introduce competitive response selection into both the bottleneck representation stage and the skip-connection fusion stage. In this way, bottleneck features can adaptively emphasize defect-related multi-scale responses, while decoder fusion can selectively balance shallow boundary details and deep semantic information. The main contributions of this paper are summarized as follows:

(1) DAMF-Net is proposed as an encoder–decoder segmentation network that introduces a unified branch-competitive modulation strategy into both bottleneck feature enhancement and skip-connection fusion. Instead of simply stacking multi-scale features or directly merging shallow and deep features, DAMF-Net formulates these two processes as adaptive feature selection problems.
(2) A Kernel Selection Fusion Attention module, termed KSFA, is designed for bottleneck-level dynamic receptive field modulation. KSFA constructs local and enlarged contextual responses through cascaded depthwise convolutions and adaptively selects scale-related feature responses by combining a spatial average prior with channel-wise Softmax competition, thereby improving the representation of defects with different scales and morphologies.
(3) An Adaptive Modulated Fusion Module, termed AMFM, is adapted for semantic-guided skip-connection fusion in steel surface defect segmentation. By introducing branch-wise Softmax competition between shallow detail features and deep semantic features, AMFM explicitly balances boundary preservation and semantic reliability during decoder fusion. This module is not claimed as a generic fusion operator independent of prior modulation designs; rather, its contribution lies in the task-oriented adaptation of competitive modulation to shallow–deep feature fusion for steel defect segmentation.
(4) Systematic experiments are conducted on the Severstal and ESDIs-SOD datasets to evaluate segmentation accuracy, robustness under complex backgrounds, and inference efficiency. The experimental results show that DAMF-Net achieves consistent performance improvements over the U-Net baseline and several representative segmentation methods, while maintaining real-time inference capability under the tested hardware setting.

## 2. Related Work

### 2.1. Research on Steel Surface Defect Recognition Based on Traditional Machine Vision and Machine Learning

Before the widespread application of deep learning, the identification of steel surface defects primarily relied on traditional machine vision and machine learning methods. Frydrych et al. [2] systematically reviewed machine vision methods in steel surface defect analysis, pointing out that these methods typically construct artificial features through edges, textures, grayscale statistics, or frequency domain information, and then use classifiers such as support vector machines or decision trees to complete defect identification. These methods can achieve certain effects in specific scenarios but are relatively sensitive to imaging conditions, background textures, and noise interference, with limited generalization ability.

Usamentiaga et al. [3] conducted a comparative analysis of object detection and semantic segmentation methods in metal surface inspection, pointing out that although traditional image processing pipelines have strong interpretability, they typically rely on significant manual tuning and experience-based design, making it difficult to adapt to the complex working conditions in the iron and steel industry, where high speeds, strong noise, and multiple defects coexist. Cheng et al. [4] further noted that traditional methods often assume controlled lighting and stable imaging, whereas perspective changes, reflections, water stains, oil pollution, and material differences in actual industrial environments can significantly reduce their stability. Therefore, traditional machine learning methods relying on handcrafted features struggle to meet the requirements of modern steel surface defect detection for robustness, real-time performance, and fine localization capabilities.

### 2.2. Research on Steel Surface Defect Segmentation Based on Deep Learning

With the development of deep learning, researchers began to utilize convolutional neural networks to automatically learn defect representations, significantly improving detection and segmentation performance in complex scenarios. U-Net, through its encoder–decoder structure and skip connections, effectively integrates shallow spatial details with deep semantic information, becoming an important foundational framework for subsequent industrial defect segmentation research [9]. On this basis, specialized networks for steel surface defect tasks continue to emerge. Ashrafi et al. [23] systematically compared the performance of various deep neural networks in steel surface defect detection and segmentation, noting that end-to-end feature learning significantly outperforms traditional manual feature methods in complex backgrounds and multi-class scenarios.

Focusing on the stable segmentation of multi-scale defects and weak defect regions, researchers have made targeted improvements to the encoder–decoder structure. Zhang et al. [24] proposed SME-DeepLabV3+ by enhancing the multi-scale enhancement strategy to improve the segmentation ability of steel surface defect regions. Yan et al. [20] proposed WPFormer, which employs a wavelet-enhanced query mechanism and a prototype-guided interaction strategy to strengthen semantic correlations in pixel-level surface defect detection, thereby improving the recognition performance for weak defects under complex background conditions. Chen and Min [25] proposed a high-precision industrial surface defect segmentation network, which improves segmentation accuracy in complex scenarios by enhancing multi-scale feature modeling and spatial information preservation mechanisms.

In addition, complex background suppression and long-range dependency modeling have gradually become new research focuses. Sun et al. [14] proposed DEPANet, which introduces an edge-guided pyramid aggregation mechanism into strip steel defect segmentation and explicitly exploits edge information to enhance multi-scale feature fusion and boundary preservation capability. Ameri and Hsu [15] proposed LACTNet, which collaboratively models local textures and global contexts using CNNs and Transformers, thereby improving the representation capability for complex defect regions. Xiao et al. [13] proposed HMNet, which introduces state-space modeling into high-resolution surface defect segmentation to enhance long-range dependency modeling and improve structural preservation in high-resolution scenarios.

Although the aforementioned deep learning-based methods have achieved significant progress in steel surface defect segmentation, several key limitations remain. First, most methods rely on fixed receptive fields or static multi-scale structures to model defects, making it difficult to simultaneously accommodate the scale differences among slender cracks, small-scale defects, and large-area structural regions, which can easily lead to scale confusion during feature extraction. Secondly, in the encoder–decoder framework, cross-layer feature fusion is typically achieved through simple concatenation or element-wise addition. Such static fusion strategies fail to effectively filter noise in shallow high-resolution features, making it easy for background interference such as reflections and texture artifacts to persist during the decoding process. Finally, although some methods introduce attention mechanisms or Transformer structures to enhance global modeling capability, their feature selection processes generally lack an explicit competition mechanism, making it difficult to achieve fine-grained selection of effective features under complex background conditions.

Therefore, how to achieve dynamic receptive field modeling in complex industrial scenarios and effectively suppress the propagation of shallow noise during cross-level fusion remains an important problem that urgently needs to be addressed.

### 2.3. Research on Lightweight and Real-Time Steel Surface Defect Segmentation

Considering the stringent requirements of industrial sites for inference speed and deployment cost, lightweight and real-time segmentation has gradually become an important direction in steel surface defect research. Zhang et al. [11] proposed CAWANet, which enhances feature representation capability while maintaining low complexity through a context-aware weighting mechanism, reflecting the design philosophy of “lightweight architecture plus context enhancement” in real-time industrial segmentation. Wang et al. [12] proposed Fast-SDNet, which pursues faster inference speed with a low parameter count from the perspective of extremely lightweight structural design, providing an effective reference for real-time steel surface defect segmentation.

In the broader direction of real-time industrial defect segmentation, Kong et al. [26] proposed PASS-Net, which achieves both high accuracy and fast inference speed on multiple industrial defect datasets through parameter-adaptive state space modeling and feature fusion modules, demonstrating that efficient global modeling and lightweight local feature extraction can achieve good synergy. Lu et al. [27] proposed DSGNet, combining depthwise separable convolutions, Ghost convolutions, and lightweight attention mechanisms into an asymmetric encoder–decoder structure, achieving good surface defect segmentation performance while maintaining low parameter counts. Zhang et al. [28] proposed FDSNeT, emphasizing the balance between accuracy and real-time performance to meet the requirements of online industrial surface defect segmentation.

In addition to specialized methods for industrial defects, general real-time segmentation studies also provide important references for steel surface defect scenarios. Yu et al. [29] proposed BiSeNet V2, which separately models spatial details and high-level semantics through a detail branch and a semantic branch, respectively, and achieves efficient fusion using a guided aggregation mechanism, thereby obtaining a favorable accuracy–speed trade-off in real-time semantic segmentation. Xu et al. [30] proposed PIDNet, which collaboratively models details, context, and boundaries through three separate pathways, further alleviating the problem that detailed features are easily overwhelmed by semantic information in real-time segmentation. These studies indicate that the core of real-time industrial segmentation is no longer merely compressing model size, but rather how to preserve effective representations of small targets, weak defects, and complex backgrounds as much as possible under limited computational resources. However, such lightweight methods usually improve inference efficiency by compressing network structures, which limits their feature representation capability in complex industrial backgrounds and makes it difficult to achieve fine-grained segmentation of both small-scale and structurally complex defects.

### 2.4. Research on Global Modeling, Multi-Scale Fusion, and Related Tasks

In addition to lightweight design, global modeling and efficient feature fusion mechanisms have gradually become important trends in visual tasks for industrial defects. Liu et al. [31] proposed VMamba, which introduces state-space models into visual backbone networks and achieves global receptive field modeling with linear complexity through two-dimensional selective scanning, providing a new technical pathway for efficient long-range dependency modeling. Although VMamba was not specifically proposed for steel surface defect segmentation, its concept of efficient global modeling offers clear inspiration for the design of industrial segmentation networks.

In steel surface defect detection tasks, many detection-oriented networks have also provided inspiration for subsequent segmentation studies. Tian and Jia [32] proposed DCC-CenterNet, which expands the receptive field through a dilated feature enhancement module and introduces an improved loss design, thereby achieving a balance between accuracy and speed in steel surface defect detection. Wang et al. [33] proposed a real-time detection network for hot-rolled steel surface defects, which improves detection accuracy and real-time performance through skip connections and pyramid feature fusion. Wei et al. [22] proposed AMFNet, which addresses the problem of increased redundant information caused by the simple fusion of high-level semantics and low-level details by designing a multi-layer feature interaction and spatial correlation modeling mechanism, thereby improving the utilization efficiency of multi-scale features in steel surface defect detection. Wu et al. [21] proposed DSAT, which combines a dynamic sparse attention mechanism with a hierarchical feature fusion structure to improve the accuracy and efficiency of detecting tiny steel surface defects. In addition, Wang et al. [34] proposed GLNet, which improves strip steel surface defect detection performance through a global–local dual-branch encoding and fusion mechanism, indicating that complementary global and local modeling is of great significance for complex industrial defect recognition.

Given that steel surface defects in complex industrial scenarios are usually accompanied by salient target–background differences, related studies on saliency detection provide useful references for defect region highlighting and boundary refinement. Song et al. [35] earlier regarded strip steel defects as salient objects and proposed a saliency detection method based on multiple constraints and improved texture features to enhance the distinguishability between defects and backgrounds. Qin et al. [36] proposed U^2^-Net, which enhances the joint modeling of high-resolution local information and low-resolution global information through a nested U-shaped structure, achieving excellent performance in salient object detection tasks. Although these methods enhance global contextual modeling capability, they still fail to explicitly consider the influence of shallow noise during cross-level propagation, and thus may still produce salient false detections under complex background conditions.

In summary, although existing methods have made certain progress in multi-scale modeling and feature fusion, they still commonly suffer from the following two key limitations: (1) multi-scale feature modeling is still dominated by fixed receptive fields or static multi-branch structures, lacking the ability to dynamically adapt to complex defect morphologies; and (2) cross-level feature fusion lacks an explicit mechanism for suppressing shallow-layer noise, which easily leads to the continuous propagation of background artifacts during the decoding stage. To address these issues, this paper presents a unified optimization design for the two key stages of the encoder and decoder from the perspective of dynamic modulation modeling.

## 3. Materials and Methods

### 3.1. Overall Architecture

**Figure 1:**
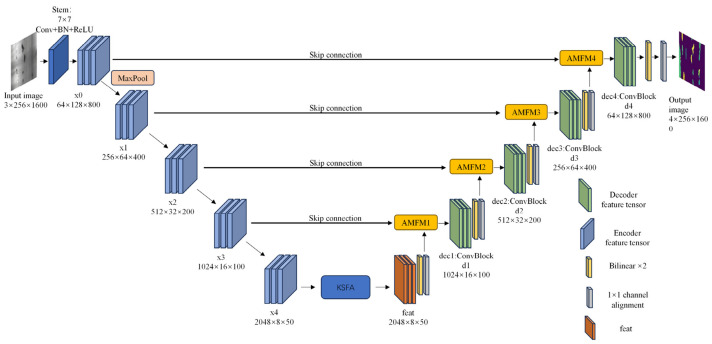
The overall framework of DAMF-Net.

To address the problems of significant scale variations, blurred defect boundaries, and interference from complex background artifacts in steel surface defect segmentation, this paper proposes an encoder–decoder defect segmentation network, termed DAMF-Net, from the perspective of dynamic modulation of feature responses and competitive selection among branches. The network adopts an encoder–decoder structure as the main framework. In the bottleneck layer of the encoder, a Kernel Selective Fusion Attention (KSFA) module is introduced to enhance the model’s adaptive representation capability for defect regions at different scales. In the decoding stage, an Adaptive Modulated Fusion Module (AMFM) is incorporated to adaptively fuse shallow high-resolution features with deep semantic features, thereby suppressing the influence of background noise during cross-level information propagation. The overall structure is shown in Figure 1.

Give an input image:

(1) $$I\in R^{B\times 3\times H\times W},$$

where B denotes the batch size, 3 represents the number of RGB color channels, and H and W denote the spatial height and width, respectively. DAMF-Net adopts ResNet-50 [37], pretrained on ImageNet, as the backbone feature extraction network after removing the fully connected layer. ResNet-50 was adopted as the encoder backbone because it provides a favorable trade-off between feature representation capability and computational cost. Its residual structure facilitates stable optimization, while ImageNet-pretrained weights provide an effective initialization for industrial defect segmentation. Moreover, the four residual stages naturally generate hierarchical features at multiple resolutions, which are well suited to the encoder–decoder framework and skip-connection fusion used in DAMF-Net. Compared with deeper backbones, ResNet-50 also reduces memory consumption, which is important for high-resolution inputs such as 256 × 1600 images in the Severstal dataset. The input image is first processed by the initial stem convolution layer and max-pooling operation, and then sequentially passed through four residual stages for feature extraction, producing multi-scale hierarchical features as follows:

(2) $$\left\{F_{1},F_{2},F_{3},F_{4}\right\},$$

where $F_{1}$, $F_{2},{\;F}_{3}\;and\;F_{4}$ correspond to spatial resolutions of 1/4, 1/8, 1/16, and 1/32 of the input image, respectively. For the ResNet-50 encoder, the tensor dimensions of the hierarchical features are expressed as follows:

(3) $$F_{1}\in R^{B\times 256\times \frac{H}{4}\times \frac{W}{4}},$$

(4) $$F_{2}\in R^{B\times 512\times \frac{H}{8}\times \frac{W}{8}},$$

(5) $$F_{3}\in R^{B\times 1024\times \frac{H}{16}\times \frac{W}{16}},$$

(6) $$F_{4}\in R^{B\times 2048\times \frac{H}{32}\times \frac{W}{32}}.$$

Additionally, the output of the Stem layer of the encoder is denoted as $F_{0}$, with 64 channels and a spatial resolution of approximately half that of the input image. These layer features contain complementary spatial and semantic information: shallow features have higher resolution, capable of preserving defect edges, textures, and local geometric details; deep features have stronger semantic abstraction capabilities, able to provide global discriminative information about defect regions.

To overcome the limitation of fixed convolutional receptive fields in adapting to complex defect scales, the KSFA module is introduced after the deepest encoder feature $F_{4}$, producing the enhanced bottleneck feature:

(7) $$F_{KSFA}=F_{KSFA}\left(F_{4}\right)\in R^{B\times 2048\times \frac{H}{32}\times \frac{W}{32}}.$$

Subsequently, the network enters the decoding and recovery stage, where the decoder progressively restores the spatial resolution through stepwise upsampling and introduces AMFM at each fusion node. Specifically, at the i-th decoding stage, the decoded feature from the previous level is first processed by bilinear upsampling and a 1 × 1 convolution outside AMFM for spatial and channel alignment, yielding the deep semantic feature $F_{deep}$. Meanwhile, the lateral skip connection from the corresponding encoder stage provides the shallow high-resolution feature $F_{shallow}$. After alignment, $F_{shallow}$ and $F_{deep}$ have the same spatial resolution and channel dimension, satisfying the input requirement of AMFM. The AMFM takes $F_{shallow}$ and $F_{deep}$ as inputs and adaptively fuses shallow details with deep semantics through Softmax-based dual-branch competitive weights for each channel. The fused feature is then fed into a basic convolutional block for spatial reconstruction. The cross-level forward propagation logic in the decoding stage can be expressed as follows:

(8) $$d_{1}=F_{\mathrm{dec}1}\left({\mathrm{AMFM}}_{1}\left(F_{3},F_{\mathrm{align}1}\left(\mathrm{Up}\left(F_{\mathrm{KSFA}}\right)\right)\right)\right)\in {\mathbb{R}}^{B\times 1024\times \frac{H}{16}\times \frac{W}{16}},$$

(9) $$d_{2}=F_{\mathrm{dec}\;2}\left({\mathrm{AMFM}}_{2}\left(F_{2},F_{\mathrm{align}\;2}\left(\mathrm{Up}\left(d_{1}\right)\right)\right)\right)\in {\mathbb{R}}^{B\times 512\times \frac{H}{8}\times \frac{W}{8}},$$

(10) $$d_{3}=F_{\mathrm{dec}\;3}\left({\mathrm{AMFM}}_{3}\left(F_{1}^{\prime },F_{\mathrm{align}\;3}\left(\mathrm{Up}\left(d_{2}\right)\right)\right)\right)\in {\mathbb{R}}^{B\times 256\times \frac{H}{4}\times \frac{W}{4}},$$

(11) $$d_{4}=F_{dec4}\left({\mathrm{AMFM}}_{4}\left(F_{0},F_{align4}\left(\mathrm{Up}\left(d_{3}\right)\right)\right)\right)\in {\mathbb{R}}^{B\times 64\times \frac{H}{2}\times \frac{W}{2}},$$

where ${AMFM}_{i}$ denotes the adaptive modulated fusion operation at the i-th decoding stage; Up(⋅) denotes the bilinear upsampling operation; $F_{align}(\cdot )$ denotes the 1×1 convolutional layer used for channel alignment; and $F_{dec}$ represents the basic convolutional reconstruction block composed of two 3×3 convolutional layers, batch normalization, and ReLU activation.

Finally, the decoder output feature $d_{4}$ is mapped to the class prediction logit tensor through the final upsampling operation and a 1×1 convolution:

(12) $$Z\in R^{B\times C\times H\times W},$$

where C denotes the number of defect categories. In the inference or probabilistic decision-making stage, an element-wise Sigmoid activation function is applied to Z to obtain the final pixel-level prediction probability map:

(13) $$P=\sigma \left(Z\right)\in R^{B\times C\times H\times W}.$$

It should be noted that the dynamic modulation strategy adopted in this paper is mainly reflected at the level of feature responses. Unlike methods that achieve dynamic modeling by altering convolutional kernel structures or spatial sampling patterns, the proposed method realizes dynamic selection of multi-scale information and cross-level features by adaptively weighting the responses of different feature branches.

For clarity, the overall forward process of DAMF-Net can be summarized as follows. First, the input image is encoded by the ResNet-50 backbone to obtain hierarchical features at different spatial resolutions. Second, the deepest encoder feature is enhanced by KSFA, where local and enlarged-context receptive-field responses are adaptively selected to obtain a scale-aware bottleneck representation. Third, during each decoding stage, the high-level decoded feature is upsampled and channel-aligned, and then fused with the corresponding shallow encoder feature through AMFM. Finally, the decoder output is mapped to the segmentation logits by a 1 × 1 convolution and restored to the input resolution. This design ensures that dynamic modulation is applied not only to the bottleneck representation but also to the cross-level feature fusion process.

### 3.2. Kernel Selection Fusion Attention Module

**Figure 2:**
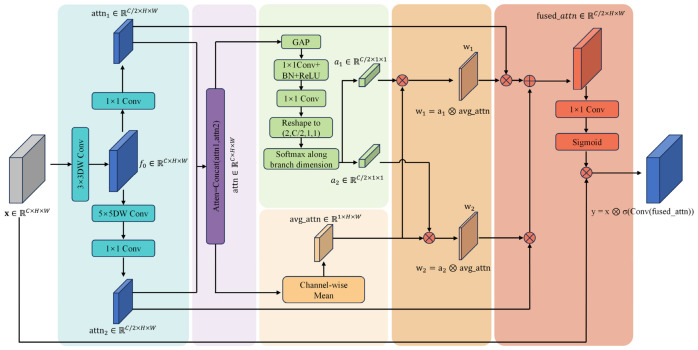
The overall architecture of KSFA.

Steel surface defects exhibit significant variations in scale and morphology, making it difficult for a single fixed convolutional receptive field to simultaneously accommodate fine scratches, slender cracks, and large-area irregular defects. Inspired by the idea of selective fusion [38], this paper designs a KSFA module at the encoder bottleneck layer, whose internal topology is shown in Figure 2. The module operates on the deepest encoder feature and generates a recalibrated bottleneck representation without changing its spatial resolution or channel dimension. This module first constructs local and relatively large-range contextual responses through a cascaded structure consisting of a 3×3 depthwise convolution and a 5×5 dilated depthwise convolution. In this design, the two receptive-field branches are not directly merged; instead, their responses are selectively modulated through channel-wise branch competition and then used to recalibrate the original bottleneck feature. It then adaptively weights the two scale branches using spatial average priors and channel-wise Softmax competitive weights, and finally recalibrates the original bottleneck features through Sigmoid gating. Unlike directly concatenating or adding multi-scale features, KSFA places greater emphasis on the selective modulation of responses at different scales, thereby improving the adaptability of deep features to complex defect morphologies.

#### 3.2.1. Cascaded Spatial Feature Extraction and Nonlinear Channel Reconstruction

Given the bottleneck feature output by the encoder:

(14) $$X\in R^{B\times C\times H\times W},$$

where C=2048. KSFA first employs a 3×3 depthwise convolution to extract local detail features $A_{ttn1}$:

(15) $$A_{ttn1}=F_{dw}^{3\times 3}\left(X\right)\in R^{B\times C\times H\times W},$$

where $F_{dw}^{3\times 3}\left(X\right)$ denotes a depthwise convolution operation with a kernel size of 3 × 3.

Subsequently, to capture contextual information over a larger range, the module takes $A_{ttn1}$ as input and cascades a depthwise convolution with a kernel size of 5×5 and a dilation rate of d=2, extracting spatial long-range features $A_{ttn2}$:

(16) $$A_{ttn2}=F_{dw,d=2}^{5\times 5}\left(A_{ttn1}\right)\in R^{B\times C\times H\times W}.$$

Since the second 5×5 convolution adopts a dilation rate of d=2, its effective kernel size is

(17) $$k_{eff}=5+\left(5-1\right)\left(d-1\right)=9.$$

Therefore, this path is formed by cascading a 3×3 depthwise convolution and a dilated depthwise convolution with an equivalent 9×9 kernel, yielding a theoretical effective receptive field of: 3 + 9 − 1 = 11, that is, an approximately 11×11 cascaded spatial receptive field. This design enlarges the perceptual range of the bottleneck layer for long-range defect structures while maintaining the low computational cost of depthwise convolution.

Afterward, the two spatial features are separately processed by independent 1×1 convolutions for channel reduction:

(18) $${\overset{∼}{A}}_{ttn1}=F_{\mathrm{conv}1}^{1\times 1}\left(A_{ttn1}\right)\in R^{B\times \frac{C}{2}\times H\times W},$$

(19) $${\overset{∼}{A}}_{ttn2}=F_{\mathrm{conv}2}^{1\times 1}\left(A_{ttn2}\right)\in R^{B\times \frac{C}{2}\times H\times W},$$

where $F_{convi}^{1\times 1}\left(\cdot \right)$ denotes a 1 × 1 convolution operation for channel transformation.

The two reduced features are concatenated along the channel dimension to obtain the joint multi-scale feature $F_{m}$:

(20) $$F_{m}=\mathrm{Concat}\left({\tilde{A}}_{ttn1},{\tilde{A}}_{ttn2}\right)\in {\mathbb{R}}^{B\times C\times H\times W}.$$

where $Concat\left(\cdot \right)$ denotes the concatenation operation along the channel dimension.

#### 3.2.2. Dynamic Weight Selection Mechanism with Decoupled Spatial and Channel Modeling

In industrial production line environments, strip steel surfaces are often accompanied by high-frequency random noise, such as random specular reflections caused by water stains or oil contamination. If extreme attention responses are directly computed over mixed spatial and channel dimensions, these irregular background artifacts can easily be introduced into the feature flow. To address this issue, KSFA adopts a dynamic generation strategy in which the spatial prior and channel weights are fully decoupled. This strategy relies on purely smooth statistical computation to enhance the overall robustness of the network against background noise.

The module first computes the element-wise average intensity response of the joint feature $F_{m}$ along the channel axis, generating a spatially smoothed prior matrix $S_{avg}$ that can capture stable spatial geometric topology:

(21) $$S_{\mathrm{avg}}={\mathrm{Mean}}_{\mathrm{channel}}\left(F_{ms}\right)\in R^{B\times 1\times H\times W}.$$

For the global channel dependency modeling branch, global average pooling is first employed to compress the joint feature $F_{ms}$, yielding the channel descriptor $Z_{ksfa}$:

(22) $$Z_{ksfa}=P_{GAP}\left(F_{ms}\right)\in R^{B\times C\times 1\times 1}.$$

Subsequently, a two-layer multilayer perceptron is introduced to capture the channel importance of multi-scale features through nonlinear channel interactions:

(23) $${\overset{\wedge }{Z}}_{ksfa}=F_{fc2}^{1\times 1}\left(\mathrm{ReLU}\left(\mathrm{BN}\left(F_{fc1}^{1\times 1}\left(Z_{ksfa}\right)\right)\right)\right)\in R^{B\times C\times 1\times 1}.$$

To achieve channel-adaptive selection between the low-level local feature branch and the high-level long-range feature branch, this paper maps the channel response tensor into a five-dimensional high-order feature space $R^{B\times 2\times \frac{C}{2}\times 1\times 1}$ with an explicit branch dimension through tensor reshaping, and applies Softmax normalization along the branch selection dimension. The weights of different branches satisfy a competitive relationship within the same channel, i.e., the sum of the branch weights is equal to 1. It should be noted that the Softmax operation is applied only along the branch dimension rather than along the spatial dimension. Therefore, KSFA performs channel-wise branch selection between receptive-field responses, instead of pixel-wise spatial selection. Accordingly, a pair of mutually exclusive channel activation weights $\left\{a_{1},a_{2}\right\}$ are derived for the two multi-scale feature branches:

(24) $$a_{1},a_{2}=S{oftmax}_{branch}({\overset{\wedge }{Z}}_{ksfa}),a_{1},a_{2}\in R^{B\times \frac{C}{2}\times 1\times 1},$$

and the bounded constraint $a_{1}+a_{2}=1$ is satisfied for any channel index.

#### 3.2.3. Dynamic Feature Adaptive Modulation and Reconstruction

After obtaining the spatial average prior $S_{avg}$ and the channel-competitive weights $a_{1}$ and $a_{2}$, KSFA multiplies them to generate the final modulation weights for the two branches:

(25) $$W_{1}=a_{1}⊗S_{\mathrm{avg}}\in R^{B\times \frac{C}{2}\times H\times W},$$

(26) $$W_{2}=a_{2}⊗S_{\mathrm{avg}}\in R^{B\times \frac{C}{2}\times H\times W},$$

where ⊗ denotes element-wise multiplication. The spatial average prior is applied to all corresponding channels via broadcasting. Subsequently, the two reduced features are weighted, modulated, and summed:

(27) $$U={\tilde{A}}_{\mathrm{ttn}1}⊗W_{1}+{\tilde{A}}_{\mathrm{ttn}2}⊗W_{2},\;\;\;\;U\in R^{B\times \frac{C}{2}\times H\times W}.$$

Finally, U is restored to the original number of channels through a 1×1 convolution, and a gating mask is generated using a Sigmoid activation function:

(28) $$G=\sigma \left(F_{\mathrm{conv}}^{1\times 1}\left(U\right)\right)\in R^{B\times C\times H\times W}.$$

The output of KSFA is given by

(29) $$F_{KSFA}=X⊗G.$$

Therefore, KSFA does not simply add multi-scale features directly. Instead, it dynamically modulates the original bottleneck feature through a combination of “cascaded dilated depthwise convolution, channel-competitive selection, spatial average prior constraint, and Sigmoid gating.” This mechanism can adaptively enhance responses related to the current defect morphology in the deep feature space while suppressing certain background interferences. Overall, KSFA follows a compact feature enhancement pipeline of multi-scale response construction, branch-wise competitive weighting, gated reconstruction, and bottleneck feature recalibration.

### 3.3. Adaptive Modulated Fusion Module

**Figure 3:**
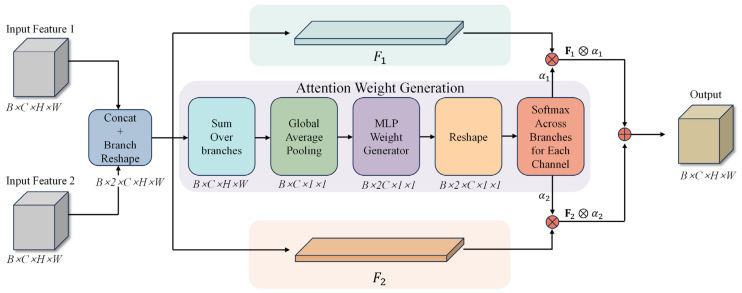
The overall architecture of the Adaptive Modulated Fusion Module.

In the encoder–decoder architecture, skip connections serve as a key pathway for fusing shallow spatial details with deep semantic information. The conventional U-Net [9] adopts channel-wise concatenation, whereas FPN [10] commonly employs element-wise addition. These fusion strategies are simple to implement, but they implicitly assume that shallow and deep features have comparable importance. However, in steel surface defect segmentation, shallow high-resolution features often contain substantial specular reflections, water stains, and texture artifacts. Without constraints from deep semantics, direct fusion can easily lead to semantic contamination, thereby degrading the final prediction quality. To address this issue, inspired by the modulation fusion strategy in image dehazing [39], this paper adapts the branch-wise competitive modulation idea to the skip-connection fusion process of steel surface defect segmentation and designs an Adaptive Modulated Fusion Module (AMFM), whose internal topology is shown in Figure 3. Different from its original use in image restoration, AMFM is reformulated in this work for semantic segmentation decoder fusion, where the two competing branches have explicit task meanings: the shallow branch provides high-resolution boundary and texture details, whereas the deep branch provides semantic guidance for defect localization. Therefore, the contribution of AMFM lies in its task-oriented adaptation to shallow–deep feature fusion in steel surface defect segmentation. AMFM constructs a joint representation of shallow detail features and deep semantic features, and introduces Softmax-normalized competitive weights along the branch dimension to achieve adaptive channel-level modulation. In this way, the model can preserve critical boundary details while suppressing shallow high-frequency noise responses.

#### 3.3.1. Cross-Level Feature Alignment and Aggregation

At the i-th decoding stage, AMFM receives two input features:

(30) $$F_{shallow}\in R^{B\times C\times H\times W},$$

(31) $$F_{deep}\in R^{B\times C\times H\times W}.$$

where $F_{shallow}$ is derived from the lateral skip connection of the encoder and contains rich spatial details, while $F_{deep}$ comes from the previous decoding stage and is spatially and channel-wise aligned through upsampling and a 1×1 convolution, containing stronger semantic information.

AMFM first concatenates the two features along the channel dimension and reshapes them into a five-dimensional tensor with an explicit branch dimension:

(32) $$F_{cat}=\mathrm{Reshape}\left(\mathrm{Concat}\left(F_{shallow\;},F_{deep}\right)\right)\in {\mathbb{R}}^{B\times 2\times C\times H\times W},$$

where $Reshape\left(\cdot \right)$ is used to introduce an explicit branch dimension for subsequent channel-wise competition modeling.

Subsequently, the two features are summed along the branch dimension to obtain a joint descriptor:

(33) $$F_{sum}=F_{shallow}⊕F_{deep}\in R^{B\times C\times H\times W},$$

where ⊕ denotes element-wise addition.

This joint descriptor is used to generate the subsequent channel-competitive weights.

#### 3.3.2. Generation of Adaptive Competitive Modulation Weights

AMFM uses global average pooling to compress the spatial dimensions of $F_{sum}$, obtaining a channel descriptor:

(34) $$Z_{MFM}=P_{GAP}\left(F_{sum}\right)\in R^{B\times C\times 1\times 1}.$$

Subsequently, a Multi-Layer Perceptron (MLP) composed of two 1×1 convolutional layers is employed to generate the weights for the two branches:

(35) $${\overset{\wedge }{Z}}_{MFM}=F_{fc2}^{1\times 1}\left(\mathrm{ReLU}\left(F_{fc1}^{1\times 1}\left(Z_{mfm}\right)\right)\right)\in R^{B\times 2C\times 1\times 1}.$$

The generated channel responses are reshaped into a tensor with an explicit branch dimension and normalized by applying Softmax along the branch dimension, yielding channel-wise branch-competitive weights:

(36) $$W_{shallow},W_{deep}={\mathrm{Softmax}}_{\mathrm{branch}}({\overset{\wedge }{Z}}_{mfm}),$$

where Softmax_branch(·) denotes a normalization operation applied along the branch dimension, enforcing a competitive relationship between the two branches, such that

(37) $$W_{shallow},W_{deep}\in R^{B\times C\times 1\times 1}.$$

This normalization process establishes a mutually exclusive competitive relationship between the two features within the same channel, satisfying

(38) $$W_{shallow}+W_{deep}=1.$$

It should be emphasized that the weights in AMFM are channel-level branch modulation weights rather than explicit pixel-wise spatial attention weights. Consequently, AMFM does not assign different fusion weights to different spatial positions directly. Instead, it learns channel-wise preferences between shallow and deep branches and applies them to the whole spatial plane by broadcasting. Their generation relies on channel descriptors obtained after global average pooling, and they are then applied to the entire spatial plane via broadcasting. Therefore, the core of AMFM lies in branch-wise competitive selection between shallow and deep features for each channel, rather than dynamic selection at the spatial-position level.

#### 3.3.3. Adaptive Modulated Fusion

After obtaining the competitive weights, the shallow and deep features were weighted and modulated separately, and their fusion was completed through element-wise weighted summation:

(39) $$F_{MFM}=\left(F_{shallow}⊗W_{shallow}\right)+\left(F_{deep}⊗W_{deep}\right)\in R^{B\times C\times H\times W}.$$

where ⊗ denotes element-wise multiplication. AMFM introduces a Softmax-based competition mechanism along the branch dimension for each channel, enabling shallow detail features and deep semantic features to be adaptively balanced and fused within the same channel. Under this mechanism, the network can learn to adjust the contributions of the two branches according to their global channel responses. When strong background noise or artifact responses are present in shallow features, the learned branch-wise competition may assign relatively higher weights to the deep semantic branch, thereby reducing the influence of shallow interference. Conversely, when boundary details are important, the shallow branch may receive relatively enhanced responses, facilitating the recovery of fine-grained structures.

Therefore, AMFM can balance structural detail preservation and noise suppression during cross-level fusion, thereby obtaining more stable and discriminative feature representations and further improving the boundary consistency and overall quality of the segmentation results.

It should be noted that AMFM is not intended to claim a new generic fusion operator independent of prior modulation fusion designs. Instead, AMFM provides a task-oriented adaptation of the branch-wise competitive modulation mechanism for the semantic segmentation decoder. In this setting, the two input branches are explicitly defined as shallow detail features and deep semantic features. Compared with the original modulation fusion design, AMFM is embedded into the skip-connection fusion nodes of the segmentation decoder to alleviate cross-level noise propagation in steel surface defect segmentation, which differs from its use in image dehazing and restoration scenarios. Overall, AMFM implements a lightweight fusion pipeline consisting of shallow–deep feature aggregation, channel descriptor generation, branch-wise Softmax competition, and weighted feature reconstruction.

### 3.4. Hybrid Loss Function and Auxiliary Supervision Strategy

Steel surface defect segmentation commonly suffers from problems such as a low proportion of defect regions, imbalanced category areas, and difficulty in recovering the boundaries of slender defects. To enhance the model’s learning capability for small-scale and low-contrast defects, this paper adopts a hybrid optimization objective that combines pixel-level binary cross-entropy loss with region-level Dice loss. Meanwhile, an auxiliary supervision head is introduced during the training stage to strengthen gradient constraints on intermediate decoding layers.

#### 3.4.1. Design of the Hybrid Loss Function

The network finally outputs the prediction logit tensor:

(40) $$Z\in R^{B\times C\times H\times W}.$$

The corresponding prediction probability is

(41) $$P=\sigma \left(Z\right).$$

Let the ground-truth label be defined as

(42) $$Y\in {\left\{0,1\right\}}^{B\times C\times H\times W}.$$

For class c, the binary cross-entropy loss is defined as

(43) $$L_{BCE}^{c}=-\frac{1}{BHW}\sum_{b=1}^{B}\sum_{h=1}^{H}\sum_{w=1}^{W}\left[Y_{b,c,h,w}\log P_{b,c,h,w}+\left(1-Y_{b,c,h,w}\right)\log\left(1-P_{b,c,h,w}\right)\right].$$

The Dice loss is defined as

(44) $$L_{\mathrm{Dice}}^{c}=1-\frac{2\sum_{b=1}^{B}\sum_{h=1}^{H}\sum_{w=1}^{W}P_{b,c,h,w}\cdot Y_{b,c,h,w}+\epsilon }{\sum_{b=1}^{B}\sum_{h=1}^{H}\sum_{w=1}^{W}P_{b,c,h,w}+\sum_{b=1}^{B}\sum_{h=1}^{H}\sum_{w=1}^{W}Y_{b,c,h,w}+\epsilon }.$$

where ϵ is a smoothing term used to prevent division by zero. The combined loss is formulated as

(45) $$L_{\mathrm{total}}^{c}=\alpha \cdot L_{BCE}^{c}+\beta \cdot L_{\mathrm{Dice}}^{c},$$

where *α* and *β* denote the weighting coefficients for the binary cross-entropy loss and the Dice loss, respectively. In this work, α and β are set to 0.5 and 0.5, respectively, so that the BCE and Dice terms contribute equally to the main prediction loss. BCE loss provides pixel-level classification supervision, while Dice loss focuses on region-level overlap and is particularly useful for defect segmentation with imbalanced foreground and background areas. Therefore, the two terms are assigned equal weights to form a balanced optimization objective without introducing excessive hyperparameter dependence.

After averaging or summing over all classes, the training loss of the main prediction branch is obtained.

#### 3.4.2. Auxiliary Supervision Strategy

In DAMF-Net, when deep supervision is enabled and the model is in the training stage, auxiliary prediction heads are introduced at two intermediate decoding layers. Specifically, the auxiliary heads are applied to $d_{3}$ and $d_{2}$, respectively, where a 1×1 convolution is used to map the features to the number of class channels, followed by bilinear interpolation to upsample them to the input image size, yielding two auxiliary prediction results:

(46) $$P_{aux}^{\left(1\right)},P_{aux}^{\left(2\right)}\in R^{B\times C\times H\times W}.$$

The total loss during the training stage can be expressed as

(47) $$L_{\mathrm{final}}=L_{\mathrm{main}}+\gamma \sum_{k=1}^{K}L_{\mathrm{aux}}^{\left(k\right)}.$$

where K=2, and γ denotes the weight of the auxiliary loss. The auxiliary loss weight γ is set to 0.4 in the reported experiments. This value is chosen to provide additional gradient guidance for intermediate decoding layers while keeping the final prediction branch dominant during optimization. Since the auxiliary heads are introduced only to regularize intermediate feature learning rather than replace the main output supervision, their contribution should be lower than that of the main loss. A relatively small auxiliary weight can help improve gradient propagation and feature discrimination in intermediate layers, while avoiding excessive interference with the final segmentation objective. It should be noted that auxiliary supervision is only effective during the training stage. In the inference stage, the model outputs only the final main prediction result; therefore, the auxiliary heads do not introduce additional computational overhead during inference.

## 4. Results

### 4.1. Experimental Settings

To verify the effectiveness of the proposed DAMF-Net in steel surface defect segmentation, experiments are conducted on two datasets, namely Severstal [40] and ESDIs-SOD [41]. The Severstal dataset is used to evaluate the model performance in multi-class steel surface defect segmentation, while the ESDIs-SOD dataset is used to validate the robustness and generalization capability of the model under complex background conditions.

#### 4.1.1. Experimental Environment Configuration

All experiments in this paper are conducted under a unified hardware and software environment, and the detailed experimental configuration is shown in Table 1.

During training, all models are trained with FP32 precision and accelerated on the GPU using CUDA. All experiments are uniformly trained for 150 epochs, with the batch size set to 2. The initial learning rate of the optimizer is set to $5\times 10^{-5}$, and the weight decay coefficient is set to $1\times 10^{-3}$. To improve training stability, a gradient clipping strategy is adopted during training, with the gradient norm limited to 1.0. The possible instability of batch normalization under small-batch training was also considered. Since all experiments were conducted on a single GPU, SyncBN was not adopted because cross-GPU synchronization of batch statistics was not applicable in the current experimental environment. Gradient accumulation was considered as a strategy to increase the effective optimization batch size. However, it does not change the per-forward-pass mini-batch statistics used by BN, and thus cannot fully eliminate the noisy statistics caused by small mini-batches. GroupNorm, which is independent of batch statistics, was further evaluated as an alternative normalization strategy. The corresponding comparison is reported in Section 4.3.3.

#### 4.1.2. Dataset Description

The Severstal dataset is a widely used public dataset for steel surface defect segmentation and contains four types of steel surface defects. Its annotations are stored in run-length encoding (RLE) format, which are decoded into four-channel binary masks in our experiments, with each channel corresponding to one defect category. Since the Severstal dataset already provides training and test sets, the original training set is further divided into a new training set and validation set at a ratio of 4:1 in this paper. The defects in this dataset exhibit large variations in scale and morphology, making it suitable for evaluating the multi-scale defect segmentation capability of the model.

The ESDIs-SOD dataset is used for salient steel surface defect detection under complex background conditions. Unlike the multi-class segmentation task in the Severstal dataset, this dataset is formulated as a binary segmentation task, where the model only needs to distinguish defect foregrounds from backgrounds. In the experiments, the ESDIs-SOD dataset is divided into training and test sets according to its official split, with an approximate ratio of 3:1. This dataset contains complex interferences such as specular reflections, water stains, oxide-scale textures, and low-contrast defects; therefore, it is mainly used to validate the robustness and generalization capability of the model under complex industrial backgrounds.

#### 4.1.3. Evaluation Metrics

To comprehensively evaluate model performance, this paper adopts Dice, mDice, IoU, mIoU, and MAE as the main segmentation evaluation metrics. Among them, Dice and IoU are used to measure the regional overlap between the predicted mask and the ground-truth mask, while MAE is used to quantify the pixel-level error between the prediction result and the ground-truth label.

The Dice coefficient is defined as follows:

(48) $${\mathrm{Dice}}_{c}=\frac{2\left|P_{c}\cap G_{c}\right|}{\left|P_{c}\right|+\left|G_{c}\right|},$$

where $P_{c}$ and $G_{c}$ denote the predicted region and the ground-truth region for class c, respectively, and ∩ represents the set intersection operation. The Dice coefficient considers both false positives and false negatives and is therefore relatively robust for defect segmentation tasks in which foreground regions occupy a small proportion.

The intersection over union (IoU) is defined as

(49) $${\mathrm{IoU}}_{c}=\frac{\left|P_{c}\cap G_{c}\right|}{\left|P_{c}\cup G_{c}\right|}.$$

For a segmentation task containing C classes, the mean intersection over union can be expressed as

(50) $$\mathrm{mIoU}=\frac{1}{C}\sum_{c=1}^{C}\frac{\left|P_{c}\cap G_{c}\right|}{\left|P_{c}\cup G_{c}\right|}.$$

where mDice and mIoU measure the segmentation performance of the model on the Severstal dataset from the perspectives of regional overlap and boundary consistency, respectively.

For the single-object salient defect detection task on ESDIs-SOD, in addition to Dice, this paper further adopts the mean absolute error (MAE) to evaluate the pixel-level deviation between the predicted probability map and the ground-truth label map. Let the predicted probability map be P=σ(Z), and the ground-truth label map be G. The MAE is defined as

(51) $$\mathrm{MAE}=\frac{1}{H\times W}\sum_{i=1}^{H}\sum_{j=1}^{W}\left|P(i,j)-G(i,j)\right|,$$

where P(i,j) and G(i,j) denote the predicted probability and the ground-truth label at pixel position (i,j), P denotes the prediction result, G denotes the ground-truth label, and H and W denote the image height and width, respectively. For the Severstal multi-class segmentation task, this paper calculates the average Dice and IoU values over all categories, namely mDice and mIoU. For the ESDIs-SOD binary segmentation task, Dice, IoU, and MAE are used for evaluation.

In addition, to evaluate the engineering applicability of the model, this paper also uses the number of parameters (Params), Floating Point Operations (FLOPs), and Frames Per Second (FPS) to analyze model complexity and real-time performance.

#### 4.1.4. Experimental Design Rationale

The experiments are designed to evaluate DAMF-Net in terms of segmentation accuracy, robustness, and module effectiveness. The Severstal dataset is used as the main benchmark because it contains four types of steel surface defects with large scale and morphological variations, making it suitable for evaluating multi-class defect segmentation and the scale-adaptive representation ability of KSFA. The ESDIs-SOD dataset is further introduced to examine robustness under complex industrial backgrounds, where reflections, stains, oxide-scale textures, and low-contrast regions may cause severe interference. This setting helps evaluate whether AMFM can reduce shallow background noise propagation during decoder fusion.

The comparison methods are selected to cover representative segmentation paradigms, including encoder–decoder baselines, context aggregation methods, attention-enhanced networks, lightweight real-time segmentation models, and recent defect-oriented methods. In addition, ablation experiments are conducted by comparing the baseline, baseline with KSFA, baseline with AMFM, and the complete DAMF-Net under the same training and evaluation protocol. Therefore, the experimental design can evaluate both the overall performance of DAMF-Net and the individual effects of its key components.

#### 4.1.5. Data Preprocessing and Augmentation

To improve experimental reproducibility and ensure a fair comparison, the preprocessing and augmentation strategy is explicitly described in this section. For the Severstal dataset, the input images and corresponding masks were processed at a resolution of 256 × 1600. The RLE annotations were decoded into four-channel binary masks, with each channel corresponding to one defect category. During training, random horizontal flipping and random vertical flipping were applied with a probability of 0.5. The same geometric transformation was synchronously applied to each image and its corresponding segmentation mask to maintain pixel-level label consistency. After augmentation, image normalization and tensor conversion were performed before the data were fed into the network.

For the ESDIs-SOD dataset, the input images and masks were processed at a resolution of 256 × 256, and the task was formulated as a binary foreground/background segmentation task. During validation and testing, only deterministic preprocessing was used, including image normalization and tensor conversion, without random data augmentation.

No random rotation, random cropping, brightness/contrast adjustment, MixUp, CutMix, or Mosaic augmentation was used in this study. This setting avoids introducing artificial boundary deformation or mixed-label artifacts, which may affect the spatial integrity of slender or low-contrast defect masks. The same preprocessing and augmentation protocol was used for DAMF-Net and all comparison models to ensure fair comparison.

### 4.2. Quantitative Comparison

To objectively evaluate the performance of DAMF-Net, this paper first conducts quantitative comparisons with various representative segmentation models on the Severstal dataset. The comparison models mainly include four categories: classical encoder–decoder architectures, such as U-Net [9], PSPNet [42], and DeepLabV3+ [43]; attention-enhanced networks, such as CBAMNet [17], PANet [44], DANet [45], and MANet [46]; lightweight and real-time segmentation networks, such as PIDNet [30], ENet [47], BiSeNetV1 [48], CAWANet [11], and Fast-SDNet [12]; and recently proposed defect segmentation methods, such as DSGNet [27], FDSNet [28], and EHMCANet [49].

#### 4.2.1. Comprehensive Performance Comparison

To verify the overall performance of DAMF-Net in multi-class steel surface defect segmentation, this paper compares it with various representative models on the Severstal dataset. This comparison is designed to evaluate whether DAMF-Net can achieve consistent improvements over different categories of segmentation models under the same dataset setting. Table 2 reports the comprehensive performance results of different methods on this dataset, where mDice and mIoU are used to measure the overall segmentation capability of the model for defect regions, and MAE is used to characterize the pixel-level error between the prediction results and the ground-truth labels.

As can be seen from Table 2, DAMF-Net achieves the best overall performance, with mDice, mIoU, and MAE values of 90.59%, 87.40%, and 0.0070, respectively. Compared to the U-Net (ResNet-50) baseline model, mDice improved by 2.38 percentage points, mIoU improved by 2.42 percentage points, and MAE decreased from 0.0076 to 0.0070, indicating that the method in this paper can effectively improve the accuracy of defect region segmentation and reduce pixel-level errors. These improvements indicate that DAMF-Net improves both region-level overlap and pixel-level prediction accuracy. The gains in mDice and mIoU suggest better defect-region coverage and boundary consistency, while the reduction in MAE indicates fewer pixel-level deviations between the predicted masks and the ground truth.

Compared to classic contextual modeling methods such as PSPNet and DeepLabV3+, DAMF-Net still demonstrates higher mDice and mIoU, indicating that relying solely on static multi-scale contextual modeling is difficult to fully adapt to the variations in scale and morphology of steel surface defects. This paper introduces the KSFA module into the encoder bottleneck layer, enhancing the model’s ability to represent multi-scale defect structures through a dynamic receptive field enhancement mechanism.

When compared with attention enhancement models, DAMF-Net also achieves further improvement compared to methods such as CBAMNet. This indicates that traditional attention mechanisms, although capable of enhancing effective feature responses, still have limited suppression of shallow background noise. DAMF-Net, through the AMFM, performs adaptive branch-wise competitive modulation for each channel between shallow detail features and deep semantic features, which helps reduce the propagation of background interference such as reflections and textures during the decoding stage.

In addition, lightweight models such as ENet, CAWANet, and Fast-SDNet, although structurally simpler, show significantly lower segmentation accuracy compared to DAMF-Net, indicating that excessive compression weakens the model’s ability to express complex defect textures and multi-scale structures. Overall, DAMF-Net achieves the highest mDice and mIoU, as well as the lowest MAE, on the Severstal dataset, validating the effectiveness of the dynamic receptive field enhancement and adaptive modulated fusion mechanism.

#### 4.2.2. Multi-Seed Statistical Validation

To further evaluate the statistical reliability and robustness of the proposed DAMF-Net, repeated experiments were conducted using five different random seeds, namely 42, 666, 1024, 2026, and 3407. For a fair comparison, the same dataset split, training protocol, hyperparameter settings, and evaluation procedure were used for both U-Net (ResNet-50) and DAMF-Net. The seed-wise mDice results on the Severstal dataset are reported in Table 3.

As shown in Table 3, DAMF-Net consistently outperforms U-Net (ResNet-50) across all five random seeds. Specifically, U-Net (ResNet-50) achieves an average mDice of 88.13 ± 0.12%, whereas DAMF-Net achieves 90.52 ± 0.09%. The mean paired improvement of DAMF-Net over U-Net (ResNet-50) is 2.39 ± 0.06 percentage points. This small standard deviation indicates that the proposed method maintains stable performance under different random initializations.

Furthermore, a paired *t*-test was performed on the five seed-wise paired results to examine whether the improvement of DAMF-Net over U-Net (ResNet-50) is statistically significant. The test result shows that the improvement is significant, with t(4) = 87.27 and *p* = 1.03 × 10^−7^. These results demonstrate that the performance gain of DAMF-Net is consistent and statistically reliable, rather than being caused by a single favorable run or random initialization.

#### 4.2.3. Fine-Grained Comparison for Individual Categories

The true challenge of industrial strip steel defects lies in the drastic morphological differences between different categories of defects. To further dissect the intrinsic behavioral patterns of the model when facing specific deformation defects, Table 4 statistically summarizes and displays the fine-grained Dice indicator across Severstal’s four independent defect categories for the compared methods.

As can be seen from Table 4, DAMF-Net achieves the best results on Class 1 and Class 3, with Dice scores of 93.54% and 74.14%, respectively. Among them, Class3 typically has characteristics such as being slender, having a large span, and high requirements for structural continuity, making it a more difficult category to segment. Compared to U-Net’s 69.05% on Class3, DAMF-Net improved by 5.09 percentage points, indicating that the method in this paper can more effectively capture long-range spatial structures and reduce the fragmentation and missed detection of slender defect areas.

For Class2 small-scale defects, DAMF-Net’s Dice reaches 98.46%, maintaining a high level. This result indicates that AMFM adaptively modulates shallow details under the guidance of deep semantics, which helps suppress background noise interference while preserving fine-grained target boundaries. For Class4 large-area irregular defects, DAMF-Net’s Dice is 96.23%, very close to the optimal result, showing that the model also has strong stability in overall structural restoration for large-scale defect areas.

Overall, DAMF-Net shows good segmentation performance on all four types of defects, with particularly significant advantages in scenarios involving slender structures and low prominence defects. This indicates that KSFA enhances the model’s ability to represent multi-scale structures through dynamic receptive field enhancement, while AMFM improves defect boundary recovery and suppresses shallow noise propagation through adaptive modulated fusion.

#### 4.2.4. Result Analysis and Discussion

From the overall experimental results, the following conclusions can be summarized:

(1) The importance of dynamic receptive field modeling: KSFA constructs a larger theoretical receptive field through cascaded depthwise convolution and dilated convolution, enabling the network to better adapt to slender cracks and large-span defect structures.
(2) Effectiveness of adaptive modulated fusion: AMFM formulates cross-level fusion as an adaptive branch-wise competitive modulation process for each channel between shallow details and deep semantics, which helps reduce the direct propagation of shallow artifacts.
(3) Improved robustness to complex backgrounds: KSFA and AMFM improve feature representation and cross-level fusion from the encoding and decoding sides, respectively, allowing DAMF-Net to achieve more stable performance in scenarios involving complex textures, specular reflections, and low-contrast defects.

Overall, the proposed method outperforms conventional approaches in both multi-scale defect modeling and adaptability to complex backgrounds, providing an effective solution for high-precision defect segmentation in industrial scenarios.

### 4.3. Ablation Experiments and Mechanism Analysis

To validate the contributions of the proposed KSFA and AMFM modules to model performance and further analyze the specific effects of different internal structural designs on segmentation results, this paper conducts systematic ablation experiments on the Severstal dataset. The U-Net (ResNet-50) model without any additional modules is used as the baseline. Under the condition that the training strategy, data augmentation scheme, and underlying hyperparameters are kept completely consistent, systematic comparative analyses are performed by progressively embedding or replacing different module variants.

#### 4.3.1. Ablation Experiments on Core Modules

To separately analyze the performance gains brought by the dynamic receptive field enhancement mechanism and the cross-level modulation fusion mechanism, this paper first conducts two groups of independent embedding experiments on the two core components of the network, and then verifies the comprehensive segmentation performance after their full integration. The quantitative results of the ablation experiments are shown in Table 5.

According to the quantitative results in Table 5, the following conclusions can be drawn. After independently introducing KSFA into the encoder bottleneck layer of the baseline model, mDice increases from 88.21% to 89.28%, mIoU increases from 84.98% to 86.05%, and MAE decreases from 0.0076 to 0.0075. This indicates that the cascaded dilated depthwise convolution and dynamic gating mechanism in KSFA can effectively enhance the representation capability of the bottleneck layer for multi-scale defect structures.

After independently introducing AMFM at the skip-connection fusion nodes, the model achieves an mDice of 89.61%, an mIoU of 86.44%, and a reduced MAE of 0.0073. Compared with the independent introduction of KSFA, AMFM brings a slightly larger improvement, indicating that adaptive cross-level feature modulation in the decoding stage plays an important role in suppressing shallow noise propagation and recovering defect boundaries.

When KSFA and AMFM are introduced simultaneously, the complete DAMF-Net achieves the best performance, with an mDice of 90.59%, an mIoU of 87.40%, and an MAE reduced to 0.0070. These results indicate that KSFA and AMFM provide compatible improvements from different network stages. KSFA mainly enhances bottleneck-level multi-scale representation, whereas AMFM improves skip-connection fusion by adaptively balancing shallow detail features and deep semantic features. Although the combined gain should not be interpreted as strict synergistic enhancement, the joint configuration achieves the best overall performance among the tested variants. Together, they improve the segmentation accuracy and robustness of the model.

The ablation experiments further show that, compared with simply enhancing feature extraction capability, adaptive modulated cross-level fusion contributes more significantly to model performance improvement. This suggests that, in complex industrial scenarios, effectively suppressing the propagation of shallow noise is more critical than merely enlarging the receptive field.

#### 4.3.2. Ablation Experiments on Internal Structural Design

To validate the rationality of the internal designs of KSFA and AMFM, fine-grained ablation experiments are conducted on different receptive-field modeling strategies and decoder fusion mechanisms. For the decoder-fusion comparison, several representative fusion strategies are considered, including direct additive fusion, channel attention-guided fusion, AFF [19], iAFF [19], sigmoid-modulated semantic fusion, and the proposed AMFM. The results are shown in Table 6.

In the comparison of multi-scale modeling strategies at the encoder bottleneck layer, the parallel multi-scale structure achieves an mDice of only 87.46%, which is lower than that of the baseline model. This indicates that simply stacking convolutional kernels of different sizes in parallel does not necessarily bring performance gains and may instead introduce redundant background responses. The cascaded multi-scale structure improves mDice to 88.74%, suggesting that progressively enlarging the receptive field is more suitable for deep defect structure modeling than simple parallel aggregation.

When the gating mechanism is removed, the performance decreases to 88.02%, indicating that merely enlarging the receptive field is insufficient to obtain stable improvements, and that dynamic gating plays an important role in selecting effective responses. The finally adopted dynamic gated receptive field enhancement structure achieves an mDice of 89.28%, demonstrating that the combination of cascaded dilated depthwise convolution and dynamic selection can more effectively enhance bottleneck-layer representation.

In the comparison of decoder fusion strategies, direct element-wise addition obtains an mDice of 87.04%, indicating that static fusion is insufficient for effective shallow–deep feature integration. Channel attention-guided fusion improves the mDice to 89.13%, showing the benefit of adaptive channel recalibration. AFF achieves an mDice of 88.19%, an mIoU of 84.95%, and an MAE of 0.0080, which is close to the baseline performance, while iAFF obtains lower mDice and mIoU values of 85.74% and 82.49%, respectively, with an MAE of 0.0081. These results suggest that generic attention-based fusion mechanisms do not necessarily bring stable improvements to decoder skip fusion in steel surface defect segmentation.

When independent Sigmoid modulation is adopted, the mDice reaches 89.47%; however, the lack of a mutually exclusive constraint may cause shallow noise and deep responses to be enhanced simultaneously. In contrast, AMFM adopts Softmax normalization along the branch dimension to establish channel-wise competition between shallow details and deep semantics. It achieves the best decoder-fusion performance, with an mDice of 89.61%, an mIoU of 86.44%, and an MAE of 0.0073. This demonstrates that AMFM is more effective in suppressing shallow background artifacts while preserving defect boundary details.

#### 4.3.3. Effect of Small-Batch Normalization Strategy

In this study, the mini-batch size was set to 2 mainly due to the high memory consumption caused by the 256 × 1600 input resolution of the Severstal dataset. Under the tested RTX 4070 SUPER GPU environment, a larger mini-batch would be memory-demanding when training DAMF-Net with the ResNet-50 encoder, decoder reconstruction blocks, KSFA, AMFM, and auxiliary prediction heads. Therefore, the same mini-batch size was used for all compared models to ensure a fair experimental setting.

Considering that Batch Normalization may produce noisy statistics under very small mini-batches, gradient clipping with a maximum norm of 1.0 was adopted during training. In addition, DAMF-Net with the original BatchNorm setting was compared with two alternatives: GroupNorm in the newly introduced decoder and modulation modules, and BatchNorm with gradient accumulation, where the effective optimization batch size was increased from 2 to 8.

SyncBN was not used because all experiments were conducted on a single GPU. Moreover, gradient accumulation increases the effective optimization batch size but does not change the per-forward-pass BatchNorm statistics. These comparisons were therefore conducted to examine the robustness of DAMF-Net under different small-batch training configurations.

As shown in Table 7, the original DAMF-Net with BatchNorm achieves an mDice of 90.59%, an mIoU of 87.40%, and an MAE of 0.0070. When GroupNorm is used, the model obtains an mDice of 90.64%, an mIoU of 87.53%, and an MAE of 0.0069, which is very close to the BatchNorm setting. When gradient accumulation is adopted, DAMF-Net achieves an mDice of 90.49%, an mIoU of 87.26%, and an MAE of 0.0071. These results indicate that DAMF-Net maintains comparable performance under different normalization and effective batch-size settings.

Overall, although the mini-batch size is small, the proposed method shows stable performance under BatchNorm, GroupNorm, and BatchNorm with gradient accumulation. This suggests that the performance improvement of DAMF-Net mainly comes from the proposed KSFA and AMFM modules rather than from a specific normalization configuration.

### 4.4. Robustness Analysis Under Complex Backgrounds

In real industrial production environments, steel surfaces are usually accompanied by complex background interferences, such as strong texture structures, nonuniform specular reflections, oxide-scale spalling, and illumination variations. To examine the robustness of the model under unknown working conditions and complex backgrounds, this paper conducts validation on the ESDIs-SOD dataset. This experiment is designed to evaluate whether different models can maintain stable defect extraction when the background contains strong visual interference and the defect foreground is weak or camouflaged.

The results of different models on the ESDIs-SOD dataset in Table 8 show that DAMF-Net achieves a Dice of 85.84%, an IoU of 76.84%, and an MAE of 0.0226, outperforming most comparison models. Compared with U-Net, DAMF-Net improves Dice and IoU by 4.40 and 5.02 percentage points, respectively, while substantially reducing MAE. The larger improvement on ESDIs-SOD than on Severstal suggests that the proposed fusion strategy is particularly beneficial under complex background interference, where shallow artifacts are more likely to be propagated through skip connections.

These results indicate that DAMF-Net not only exhibits favorable geometric fitting capability in the multi-class defect segmentation task on Severstal, but also maintains strong anti-interference capability in salient defect detection under complex background conditions. Its advantages mainly come from two aspects. First, KSFA expands the receptive field through cascaded dilated depthwise convolution in the bottleneck layer and regulates the original feature response via dynamic gating, which helps enhance the contextual perception ability for complex defect shapes. Second, AMFM performs Softmax-based competitive modulation between shallow details and deep semantics at skip connections during the decoding stage, which can reduce the impact of shallow artifacts such as reflections, stains, and scale textures on the final prediction to some extent.

Therefore, DAMF-Net shows lower pixel-level errors and more stable defect region extraction capabilities in complex backgrounds. This indicates that the method in this paper has certain cross-scenario generalization ability and can maintain stable performance under unseen distribution conditions.

### 4.5. Qualitative and Interpretability Analysis

In the field of industrial vision inspection, relying solely on macro quantitative indicators cannot fully reflect the actual behavioral patterns of the model when facing complex backgrounds and multi-scale deformation defects. To more intuitively verify the visual representation advantages of DAMF-Net in fine-grained defect recognition, boundary preservation, and background noise suppression, this section conducts qualitative analysis of the segmentation characteristics of different models through visualizing prediction results.

#### 4.5.1. Qualitative Comparison on the Severstal Dataset

**Figure 4:**
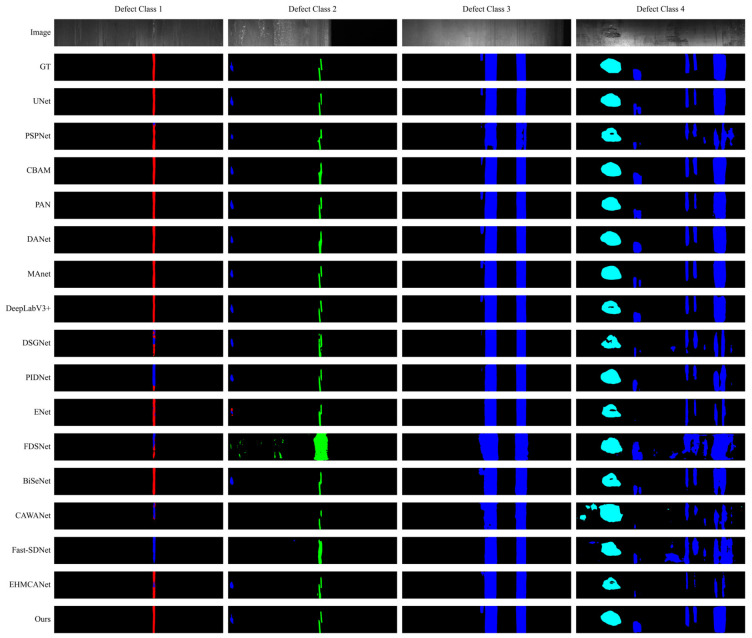
Comparison of predicted masks of different models on the Severstal dataset.

As shown in Figure 4, the visualization results on the Severstal dataset indicate that DAMF-Net can well fit the real defect regions on all four types of defect samples. For slender-type and locally structurally complex defects, the model can maintain a relatively continuous main defect structure; for small-scale low prominence defects, the model can accurately locate the core defect region; for large-scale irregular exfoliation defects, the model can relatively completely restore the target region while reducing false positives in the surrounding background.

These results indicate that expanding the receptive field in the bottleneck layer helps us to improve the model’s ability to capture long-range defect structures, while the adaptive modulated fusion performed by AMFM during the decoding stage helps us to suppress shallow noise while restoring structural details.

#### 4.5.2. Qualitative Comparison and Analysis on the ESDIs-SOD Dataset

**Figure 5:**
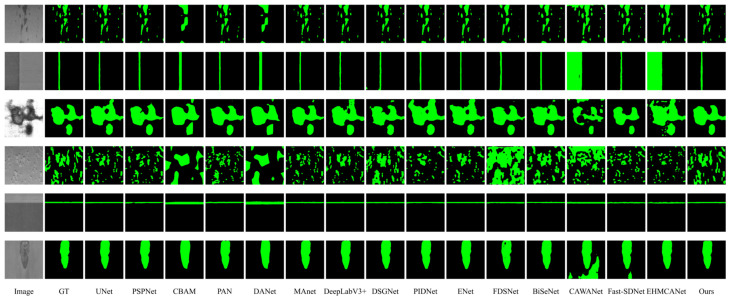
Comparison of predicted masks of different models on the ESDIs-SOD dataset.

To further examine the upper limit of the model’s anti-interference capability under extreme noise conditions, Figure 5 presents the qualitative extraction results of the compared models on the ESDIs-SOD dataset. Compared with the multi-class Severstal dataset, the strip steel backgrounds in this scenario contain numerous intertwined interferences, including specular reflections from water stains, emulsion droplets, and nonuniform oxide-scale textures, which can easily induce false-positive detections in conventional networks.

According to the visualized masks in Figure 5, on extreme samples with complex background texture structures or extremely low target contrast, methods such as CAWANet, Fast-SDNet, and some multi-scale contextual comparison methods exhibit obvious false-positive expansion in background regions, with their prediction results containing a large number of scattered redundant masks unrelated to the true defects.

In contrast, DAMF-Net demonstrates highly stable prediction performance under various strong-noise conditions: for large-area salient targets and strong-reflection samples, the outputs of DAMF-Net are more compact, enabling accurate recovery of defect contours while avoiding excessive boundary expansion, thereby exhibiting favorable mask purity and background suppression capability. For low-contrast and camouflaged weak-saliency samples, when the targets tend to be hidden, some comparison models are prone to large-area target discontinuities or confusion caused by adhesion between the target and background; however, benefiting from the channel-level Softmax competition mechanism of AMFM in the decoding stage, DAMF-Net introduces strong guidance from deep high-level semantics and can effectively avoid semantic contamination caused by spatial high-frequency reflection noise, thereby preserving the overall main structure of the defect targets more completely.

#### 4.5.3. Summary of Qualitative and Interpretability Analysis

Based on the visualization results on both the Severstal and ESDIs-SOD datasets, DAMF-Net demonstrates favorable prediction stability across different types of defects and complex background conditions. Compared with the competing models, the proposed method exhibits superior performance in terms of defect-region integrity, boundary preservation, and background noise suppression, indicating that it not only achieves favorable quantitative results but also shows strong segmentation quality and scene adaptability at the visual level.

The interpretability of DAMF-Net can be further understood from the explicit branch meanings in the proposed KSFA and AMFM modules. In KSFA, the two branches correspond to local receptive-field responses and enlarged contextual receptive-field responses, respectively. The Softmax competition along the branch dimension therefore reflects the model’s adaptive preference for different receptive-field responses. For fine scratches and small-scale defects, local responses help preserve detailed structural information, whereas for elongated cracks and large-area irregular defects, enlarged contextual responses are beneficial for capturing more continuous spatial structures. This interpretation is consistent with the class-wise results on the Severstal dataset, where DAMF-Net achieves clear improvement on structurally challenging defect categories.

In AMFM, the two competing branches have explicit semantic meanings: the shallow branch provides high-resolution boundary and texture details, while the deep branch provides stronger semantic guidance for defect localization. Since shallow features may contain background artifacts such as specular reflections, stains, and oxide-scale textures, direct skip-connection fusion can easily introduce noise into the decoder. By contrast, the branch-wise Softmax competition in AMFM enables the decoder to adaptively balance shallow details and deep semantics for each channel. When shallow features are contaminated by background interference, the deep semantic branch can provide more reliable guidance to reduce false-positive responses; when boundary recovery is important, useful shallow details can still be preserved.

Therefore, the proposed modules improve interpretability by converting multi-scale representation and skip-connection fusion into explicit branch-wise selection processes. KSFA explains how the model adaptively selects receptive-field responses for defects with different scales and morphologies, while AMFM explains how the decoder balances semantic reliability and boundary detail preservation under complex backgrounds. Together with the ablation results, these observations suggest that the performance improvement of DAMF-Net is not merely caused by increased model capacity, but is closely related to the designed dynamic modulation behavior.

### 4.6. Model Complexity and Real-Time Performance Analysis

In practical industrial applications, a model is required not only to achieve high segmentation accuracy but also to satisfy certain computational efficiency requirements. Therefore, this paper analyzes the efficiency performance of the proposed model from three aspects: the number of parameters (Params), computational complexity (GFLOPs), and inference speed (FPS).

As shown in Table 9, DAMF-Net has 58.54 M parameters, a computational cost of 146.48 GFLOPs, and an inference speed of 55.8 FPS. Although its parameter count and computational cost are higher than those of some extremely lightweight models, its inference speed still exceeds the real-time detection threshold of 30 FPS, indicating a certain degree of feasibility for engineering deployment.

From the structural perspective, the computational overhead of DAMF-Net mainly comes from the ResNet-50 encoder, decoder convolutional blocks, and the bottleneck KSFA module. KSFA constructs multi-scale receptive fields using depthwise convolutions, which can reduce the growth of parameters and computation compared with standard convolutions. AMFM is mainly composed of global average pooling, lightweight 1 × 1 convolutions, and branch-wise Softmax normalization, introducing relatively limited additional overhead. The auxiliary supervision heads are enabled only during the training stage and do not participate in the final forward output during inference; therefore, they do not increase the computational burden during deployment.

Based on the results of both accuracy and efficiency, DAMF-Net does not pursue extreme lightweight design. Instead, it achieves a balance among high segmentation accuracy, robustness to complex backgrounds, and real-time inference capability. Although the computational complexity of DAMF-Net is higher than that of some lightweight models, its clear improvements in segmentation accuracy and robustness make it highly valuable for practical industrial applications.

## 5. Discussion

### 5.1. Analysis of the Dynamic Modulation Modeling Mechanism

The experimental results indicate that the proposed dynamic modulation strategy is effective for steel surface defect segmentation under the evaluated settings. According to the ablation results, introducing KSFA at the encoder bottleneck improves the baseline performance, suggesting that adaptive receptive-field response selection is helpful for representing defects with different scales and morphologies. Introducing AMFM at the decoder fusion nodes also improves the baseline, indicating that adaptive shallow–deep feature fusion can reduce the negative influence of shallow background artifacts during reconstruction.

From a modeling perspective, KSFA and AMFM can be understood as two branch-wise competitive selection mechanisms applied at different stages of the encoder–decoder framework. KSFA focuses on selecting local and enlarged-context receptive-field responses at the bottleneck layer, whereas AMFM focuses on balancing shallow detail features and deep semantic features during skip-connection fusion. The joint configuration achieves the best performance among the tested variants, but this result should be interpreted as compatible improvements from different network stages rather than strict synergistic enhancement.

The interpretability analysis further supports this interpretation. The branch-weight behavior of KSFA suggests that the model can adjust receptive-field preferences according to defect morphology, while the shallow/deep branch weights in AMFM indicate that the decoder can adaptively balance semantic reliability and boundary detail preservation. These observations provide additional evidence for the effectiveness of the proposed modulation strategy.

### 5.2. Analysis of Method Advantages and Applicability

The advantages of DAMF-Net are mainly reflected in three aspects. First, the results on the Severstal dataset show that DAMF-Net improves multi-class defect segmentation performance compared with the U-Net baseline and several representative segmentation models. The category-level comparison further indicates that the proposed method is beneficial for structurally complex defects, especially categories requiring broader contextual perception.

Second, the results on the ESDIs-SOD dataset show that DAMF-Net maintains stable segmentation performance under complex background interference. Compared with the Severstal dataset, ESDIs-SOD contains stronger visual disturbances, such as reflections, stains, and oxide-scale textures. The improved Dice, IoU, and MAE on this dataset suggest that the proposed adaptive fusion strategy is useful for reducing the influence of shallow background artifacts in such scenarios.

Third, the complexity analysis shows that DAMF-Net maintains real-time inference capability under the tested hardware setting, although its parameter count and computational complexity are higher than those of extremely lightweight models. Therefore, DAMF-Net is more suitable for industrial scenarios where segmentation accuracy and robustness are prioritized while moderate computational resources are available.

### 5.3. Analysis of Limitations

Although DAMF-Net achieves improved segmentation accuracy and robustness in the reported experiments, several limitations remain. First, the model adopts ResNet-50 as the backbone and introduces modulation modules in both the encoder and decoder. As a result, its parameter count and computational complexity are higher than those of extremely lightweight segmentation networks. Although the measured FPS still satisfies real-time inference under the tested hardware setting, deployment on resource-constrained embedded devices may require further model compression or lightweight backbone design.

Second, the current model mainly relies on convolutional feature extraction and channel-wise branch modulation. Although KSFA enlarges the effective receptive field at the bottleneck layer, the ability to model ultra-long-range dependencies is still limited compared with recent Transformer- or state-space-based architectures. This limitation may affect performance in cases involving very large crossed defects, long-range structural discontinuities, or severe global background variations.

Third, the experiments were mainly conducted on public datasets. Although Severstal and ESDIs-SOD provide useful benchmarks for multi-class defect segmentation and complex-background evaluation, real production lines may involve additional variations in imaging devices, illumination conditions, steel types, and defect distributions. Therefore, further validation on more real-world industrial data is still necessary before large-scale deployment.

Fourth, the weighting coefficients in the hybrid loss and auxiliary supervision strategy are empirically fixed in this study. Although the adopted setting follows the principle of balancing pixel-level BCE supervision, region-level Dice supervision, and relatively weaker auxiliary constraints, a systematic sensitivity analysis of these coefficients has not been fully explored. Different defect datasets or different foreground-background imbalance levels may require slightly different loss-weight configurations. In future work, adaptive loss weighting or uncertainty-based dynamic weighting strategies will be investigated to further reduce manual hyperparameter dependence.

## 6. Conclusions and Future Work

This paper proposes DAMF-Net for steel surface defect segmentation under scale variation and complex background interference. The proposed method introduces KSFA at the encoder bottleneck to enhance scale-adaptive feature representation and AMFM in the decoder to adaptively fuse shallow detail features and deep semantic features. In this way, multi-scale bottleneck representation and skip-connection fusion are both formulated as branch-wise competitive modulation processes.

Experimental results on the Severstal dataset show that DAMF-Net improves multi-class steel defect segmentation performance compared with the U-Net baseline and several representative segmentation models. The category-level results further indicate that the proposed method is effective for defects with complex structures and large morphological variations. On the ESDIs-SOD dataset, DAMF-Net also achieves improved Dice, IoU, and MAE under complex background conditions, suggesting better robustness to visual interference such as reflections, stains, and oxide-scale textures.

The ablation experiments verify the individual effectiveness of KSFA and AMFM. KSFA improves bottleneck-level feature representation through adaptive receptive-field response selection, while AMFM improves decoder fusion by balancing shallow details and deep semantic guidance. The joint configuration achieves the best performance among the tested variants, but the gain is interpreted as compatible improvements from different network stages rather than strict synergistic enhancement. In addition, the branch-weight analysis provides further evidence that KSFA and AMFM perform adaptive response selection during feature enhancement and feature fusion.

Despite these improvements, DAMF-Net still has limitations. Its computational complexity is higher than that of extremely lightweight segmentation networks, and its global dependency modeling capability remains limited by the mainly convolutional architecture. Future work will focus on designing lighter dynamic modulation modules, exploring Transformer or state-space models for long-range dependency modeling, and validating the proposed framework on more real-world industrial production data.

## Figures and Tables

**Table 1:** Hardware and software configuration of the experimental environment.

Configuration Item	Specific Configuration
Operating system	Windows 11
Central processing unit	Intel Core i5-14600KF
Random access memory	DDR5 16 GB × 2
Graphics processing unit	RTX 4070 SUPER
Deep learning framework	PyTorch 2.5.1
CUDA	CUDA 12.1
Programming language	Python 3.10.19

**Table 2:** Quantitative comparison of different segmentation methods on the Severstal dataset.

Method	mDice%	mIoU%	MAE
U-Net (ResNet-50)	88.21	84.98	0.0076
PSPNet	87.56	84.21	0.0092
DeepLabV3+	89.58	86.42	0.0078
CBAMNet	90.11	86.79	0.0079
PANet	87.23	83.94	0.0084
DANet	89.48	86.19	0.0079
MAnet	88.33	85.15	0.0072
PIDNet	86.87	83.59	0.0095
ENet	82.53	79.25	0.0083
BiSeNetV1	88.04	84.78	0.0079
CAWANet	76.24	72.84	0.0174
Fast-SDNet	80.54	77.14	0.0102
DSGNet	83.45	80.08	0.0090
FDSNet	83.64	80.27	0.0109
EHMCANet	85.73	82.41	0.0091
DAMF-Net(Ours)	90.59	87.40	0.0070

**Table 3:** Multi-seed mDice comparison between U-Net (ResNet-50) and DAMF-Net on the Severstal dataset.

Seed	U-Net (ResNet-50)	DAMF-Net
42	0.8821	0.9059
666	0.8796	0.9043
1024	0.8806	0.9047
2026	0.8824	0.9063
3407	0.8818	0.9048
Mean ± std	88.13 ± 0.12	90.52 ± 0.09

**Table 4:** Class-wise Dice comparison of different models on the Severstal dataset.

Method	Class 1	Class 2	Class 3	Class 4
U-Net (ResNet-50)	91.65	98.20	69.05	93.94
PSPNet	92.72	98.5	64.15	94.87
DeepLabV3+	92.40	98.22	72.64	95.05
CBAMNet	92.73	98.34	73.11	96.26
PANet	90.82	98.37	66.63	93.11
DANet	92.28	98.22	71.75	95.69
MAnet	88.12	98.27	70.77	96.18
PIDNet	86.83	98.25	66.89	95.52
ENet	79.31	93.35	64.46	92.99
BiSeNetV1	91.34	98.14	68.66	94.02
CAWANet	70.94	89.99	56.79	87.25
Fast-SDNet	74.92	98.34	59.51	89.40
DSGNet	82.76	93.84	64.20	93.07
FDSNet	81.06	97.41	65.03	91.06
EHMCANet	82.61	98.25	67.64	94.43
DAMF-Net (Ours)	93.54	98.46	74.14	96.23

**Table 5:** Ablation study of the main components of DAMF-Net on the Severstal dataset.

Baseline	KSFA	AMFM	mDice%	mIoU%	MAE
✓			88.21	84.98	0.0076
✓	✓		89.28	86.05	0.0075
✓		✓	89.61	86.44	0.0073
✓	✓	✓	90.59	87.40	0.0070

Note: ✓ Indicates the network where this module exists.

**Table 6:** Ablation study of internal designs in KSFA and AMFM.

Method	mDice (%)	mIoU (%)	MAE
Baseline	88.21	84.98	0.0076
Baseline + Parallel multiscale receptive field modeling	87.46	84.27	0.0079
Baseline + Cascaded multiscale receptive field modeling	88.74	85.55	0.0075
Baseline + Residual gated receptive field enhancement	88.88	85.75	0.0076
Baseline + Cascaded multiscale receptive field modeling w/o gating	88.02	84.79	0.0076
Baseline + KSFA	89.28	86.05	0.0075
Baseline + Direct additive fusion	87.04	83.77	0.0079
Baseline + Channel attention-guided fusion	89.13	85.90	0.0076
Baseline + AFF	88.19	84.95	0.0080
Baseline + iAFF	85.74	82.49	0.0081
Baseline + Sigmoid-modulated semantic fusion	89.47	86.25	0.0074
Baseline + AMFM	89.61	86.44	0.0073
Baseline + KSFA + AMFM	90.59	87.40	0.0070

**Table 7:** Effect of normalization and effective batch-size strategies on the Severstal dataset.

Method	Mini-Batch Size	Effective Batch Size	Normalization Setting	mDice%	mIoU%	MAE
DAMF-Net	2	2	BN	90.59	87.40	0.0070
DAMF-Net	2	2	GroupNorm	90.64	87.53	0.0069
DAMF-Net	2	8	BN + gradient accumulation	90.49	87.26	0.0071

**Table 8:** Robustness comparison of different models on the ESDIs-SOD dataset under complex background conditions.

Method	Dice%	IoU%	MAE
U-Net	81.44	71.82	0.0404
PSPNet	76.74	66.36	0.0388
DeepLabV3+	82.63	73.38	0.0285
CBAMNet	70.45	59.04	0.0509
PANet	82.66	72.95	0.0338
DANet	68.72	57.27	0.0546
MAnet	85.15	76.04	0.0268
PIDNet	78.91	68.30	0.0413
ENet	81.99	72.41	0.0292
BiSeNetV1	82.58	72.59	0.0335
CAWANet	57.55	46.82	0.2059
Fast-SDNet	81.35	71.62	0.0360
DSGNet	81.56	71.74	0.0339
FDSNet	78.06	67.43	0.0686
EHMCANet	61.84	48.74	0.4829
DAMF-Net(Ours)	85.84	76.84	0.0226

**Table 9:** Comparison of model complexity and inference speed among different models.

Model	Params (M)	GFLOPs	FPS
U-Net (ResNet-50)	32.52	66.89	93.5
PSPNet	24.31	18.46	265.2
CBAM-Net	24.04	33.56	181.1
PAN	24.26	54.37	92.9
DANet	47.63	46.93	139.2
MAnet	147.44	116.88	56.7
DeepLabV3+	26.68	57.41	117.7
DSGNet	0.49	3.15	107.5
PIDNet	7.62	9.27	176.6
ENet	0.35	3.03	106.0
FDSNet	0.96	1.62	319.2
BiSeNet	12.43	19.09	296.9
CAWANet	0.48	1.41	371.0
Fast-SDNet	0.07	0.94	261.8
EHMCANet	24.59	484.19	15.9
DAMF-Net (Ours)	58.54	146.48	55.8

## Data Availability

The original contributions presented in this study are included in the article. Further inquiries can be directed to the corresponding author.

## Abbreviations

The following abbreviations are used in this manuscript:

KSFA	Kernel Selective Fusion Attention
AMFM	Adaptive Modulated Fusion Module
RLE	Run-length encoding
MLP	Multi-Layer Perceptron
MAE	Mean absolute error

## References

[1] Bai D., Li G., Jiang D., Tao B., Yun J., Hao Z., Zhou D., Ju Z. (2024). Depth Feature Fusion Based Surface Defect Region Identification Method for Steel Plate Manufacturing. Comput. Electr. Eng..

[2] Frydrych K., Tomczak M., Jasiński J., Papanikolaou S. (2025). Steel Surface Defects Analysis with Machine Vision and Deep Learning. Int. J. Adv. Manuf. Technol..

[3] Usamentiaga R., Lema D.G., Pedrayes O.D., Garcia D.F. (2022). Automated Surface Defect Detection in Metals: A Comparative Review of Object Detection and Semantic Segmentation Using Deep Learning. IEEE Trans. Ind. Appl..

[4] Cheng Y., Cao Y., Yao H., Luo W., Jiang C., Zhang H., Shen W. (2026). A Comprehensive Survey for Real-World Industrial Surface Defect Detection: Challenges, Approaches, and Prospects. J. Manuf. Syst..

[5] Xu M., Wei J., Feng X. (2024). Two-Stage Encoder Multi-Decoder Network with Global–Local up-Sampling for Defect Segmentation of Strip Steel Surface Defects. Eng. Appl. Artif. Intell..

[6] Wang J., Long Z., Lee M.-J., Feng Y., E J., Xu Y., Zhang Y., Alexander I., Tang X., Fan R. (2025). Edge-Aware and Deformable Feature Fusion for Steel Surface Defect Segmentation. Proceedings of the 2025 IEEE International Conference on Real-Time Computing and Robotics (RCAR).

[7] Li M., Liu Y., Chen D., Li X. (2025). Lightweight Metal Surface Defect Segmentation Method Based on Multi-Scale Feature Fusion and Knowledge Distillation. J. Supercomput..

[8] Luo Q., Li B., Su J., Yang C., Gui W., Silvén O., Liu L. (2024). CDDNet: Camouflaged Defect Detection Network for Steel Surface. IEEE Trans. Instrum. Meas..

[9] Ronneberger O., Fischer P., Brox T. (2015). U-Net: Convolutional Networks for Biomedical Image Segmentation. Proceedings of the International Conference on Medical Image Computing and Computer-Assisted Intervention (MICCAI).

[10] Lin T.-Y., Dollár P., Girshick R., He K., Hariharan B., Belongie S. (2017). Feature Pyramid Networks for Object Detection. Proceedings of the IEEE Conference on Computer Vision and Pattern Recognition.

[11] Zhang G., Lu Y., Jiang X., Yan F., Xu M. (2024). Context-Aware Adaptive Weighted Attention Network for Real-Time Surface Defect Segmentation. IEEE Trans. Instrum. Meas..

[12] Wang R., Zheng Y., He Z., Wang H., Ren W., Zuo H. (2025). Fast-SDNet: An Extremely Lightweight and Accurate Surface Defect Segmentation Network. Nondestruct. Test. Eval..

[13] Xiao H., Ren Z., Cai S. (2025). HMNet: High-Resolution Mamba Network for Semantic Segmentation of Surface Defects. Proceedings of the 2025 28th International Conference on Computer Supported Cooperative Work in Design (CSCWD).

[14] Sun Y., Geng S., Zheng C., Xu C., Guo H., Feng Y. (2025). DEPANet: A Differentiable Edge-Guided Pyramid Aggregation Network for Strip Steel Surface Defect Segmentation. Algorithms.

[15] Ameri R., Hsu C.-C. (2026). LACTNet: A Label-Aware CNN-Transformer Network for Surface Defect Segmentation. Expert Syst. Appl..

[16] Hu J., Shen L., Albanie S., Sun G., Wu E. (2019). Squeeze-and-Excitation Networks. Proceedings of the IEEE Conference on Computer Vision and Pattern Recognition.

[17] Woo S., Park J., Lee J.-Y., Kweon I.S. (2018). CBAM: Convolutional Block Attention Module. Proceedings of the European Conference on Computer Vision (ECCV).

[18] Li X., Wang W., Hu X., Yang J. (2019). Selective Kernel Networks. Proceedings of the 2019 IEEE/CVF Conference on Computer Vision and Pattern Recognition (CVPR).

[19] Dai Y., Gieseke F., Oehmcke S., Wu Y., Barnard K. (2021). Attentional Feature Fusion. Proceedings of the 2021 IEEE Winter Conference on Applications of Computer Vision (WACV).

[20] Yan F., Jiang X., Lu Y., Cao J., Chen D., Xu M. (2025). Wavelet and Prototype Augmented Query-Based Transformer for Pixel-Level Surface Defect Detection. Proceedings of the Computer Vision and Pattern Recognition Conference.

[21] Wu S., Yang H., Liao L., Song C., Fang Y., Fu J., Li T. (2025). DSAT: A Dynamic Sparse Attention Transformer for Steel Surface Defect Detection with Hierarchical Feature Fusion. Sci. Rep..

[22] Wei C., Bao Y., Zheng C., Ji Z. (2026). AMFNet: Aggregated Multi-Level Feature Interaction Fusion Network for Defect Detection on Steel Surfaces. J. Intell. Manuf..

[23] Ashrafi S., Teymouri S., Etaati S., Khoramdel J., Borhani Y., Najafi E. (2025). Steel Surface Defect Detection and Segmentation Using Deep Neural Networks. Results Eng..

[24] Zhang H., Zhao Z., Liu Y., Liu J., Ma T., Wu K., Zhuang Z., Wang J. (2025). Steel Surface Defect Segmentation with SME-DeeplabV3+. PLoS ONE.

[25] Chen H., Min B.-W. (2025). A High-Precision Segmentation Network for Industrial Surface Defect Detection. AIP Adv..

[26] Kong L., Mei J., Jia F., Du W. (2026). PASS-Net: Parameter-Adaptive State-Space Network for Real-Time and High-Accuracy Segmentation of Industrial Surface Defects. Appl. Soft Comput..

[27] Lu H., Zhao Y., Yang G., Wu S. (2026). DSGNet: A Lightweight Network Integrating Depthwise Separable and Ghost Convolutions for Real-time Surface Defect Segmentation. Expert Syst..

[28] Zhang J., Ding R., Ban M., Guo T. (2022). FDSNeT: An Accurate Real-Time Surface Defect Segmentation Network. Proceedings of the ICASSP 2022—2022 IEEE International Conference on Acoustics, Speech and Signal Processing (ICASSP).

[29] Yu C., Gao C., Wang J., Yu G., Shen C., Sang N. (2021). BiSeNet V2: Bilateral Network with Guided Aggregation for Real-Time Semantic Segmentation. Int. J. Comput. Vis..

[30] Xu J., Xiong Z., Bhattacharyya S.P. (2023). PIDNet: A Real-Time Semantic Segmentation Network Inspired by PID Controllers. Proceedings of the IEEE/CVF Conference on Computer Vision and Pattern Recognition.

[31] Liu Y., Tian Y., Zhao Y., Yu H., Xie L., Wang Y., Ye Q., Jiao J., Liu Y. (2024). VMamba: Visual State Space Model. Adv. Neural Inf. Process. Syst..

[32] Tian R., Jia M. (2022). DCC-CenterNet: A Rapid Detection Method for Steel Surface Defects. Measurement.

[33] Wang W., Mi C., Wu Z., Lu K., Long H., Pan B., Li D., Zhang J., Chen P., Wang B. (2022). A Real-Time Steel Surface Defect Detection Approach with High Accuracy. IEEE Trans. Instrum. Meas..

[34] Wang X., Bao L., Zhou X., Xia L., Xu X. (2025). GLNet: Global-Local Fusion Network for Strip Steel Surface Defects Detection. IEEE Signal Process. Lett..

[35] Song G., Song K., Yan Y. (2020). Saliency Detection for Strip Steel Surface Defects Using Multiple Constraints and Improved Texture Features. Opt. Lasers Eng..

[36] Qin X., Zhang Z., Huang C., Dehghan M., Zaiane O.R., Jagersand M. (2020). U2-Net: Going Deeper with Nested U-Structure for Salient Object Detection. Pattern Recognit..

[37] He K., Zhang X., Ren S., Sun J. (2015). Deep Residual Learning for Image Recognition. Proceedings of the IEEE Conference on Computer Vision and Pattern Recognition (CVPR).

[38] Xu Y., Wang D., Zhang L., Zhang L. (2025). Dual Selective Fusion Transformer Network for Hyperspectral Image Classification. Neural Netw..

[39] Zhang Y., Zhou S., Li H. (2024). Depth Information Assisted Collaborative Mutual Promotion Network for Single Image Dehazing. Proceedings of the 2024 IEEE/CVF Conference on Computer Vision and Pattern Recognition (CVPR).

[40] Kaggle (2019). Severstal Steel Defect Detection. https://www.Kaggle.Com/c/Severstal-Steel-Defect-Detection.

[41] Cui W., Song K., Feng H., Jia X., Liu S., Yan Y. (2023). Autocorrelation-Aware Aggregation Network for Salient Object Detection of Strip Steel Surface Defects. IEEE Trans. Instrum. Meas..

[42] Zhao H., Shi J., Qi X., Wang X., Jia J. (2017). Pyramid Scene Parsing Network. Proceedings of the 2017 IEEE Conference on Computer Vision and Pattern Recognition (CVPR).

[43] Chen L.-C., Zhu Y., Papandreou G., Schroff F., Adam H., Ferrari V., Hebert M., Sminchisescu C., Weiss Y. (2018). Encoder-Decoder with Atrous Separable Convolution for Semantic Image Segmentation. Computer Vision—ECCV 2018.

[44] Li H., Xiong P., An J., Wang L. (2018). Pyramid Attention Network for Semantic Segmentation. arXiv.

[45] Fu J., Liu J., Tian H., Li Y., Bao Y., Fang Z., Lu H. (2019). Dual Attention Network for Scene Segmentation. Proceedings of the IEEE/CVF Conference on Computer Vision and Pattern Recognition (CVPR).

[46] Fan T., Wang G., Li Y., Wang H. (2020). MA-Net: A Multi-Scale Attention Network for Liver and Tumor Segmentation. IEEE Access.

[47] Paszke A., Chaurasia A., Kim S., Culurciello E. (2016). ENet: A Deep Neural Network Architecture for Real-Time Semantic Segmentation. arXiv.

[48] Yu C., Wang J., Peng C., Gao C., Yu G., Sang N. (2018). BiSeNet: Bilateral Segmentation Network for Real-Time Semantic Segmentation. Proceedings of the European Conference on Computer Vision (ECCV 2018).

[49] Wang B., Wei Z., Ju M., Zhao Z., Zhang S. (2025). Efficient Hierarchical Multiscale Convolutional Attention for Accurate Medical Image Segmentation. Vis. Comput..

